# DeepCYP: an integrated deep learning web server for the holistic “pathway-site product” prediction of CYP450 metabolism

**DOI:** 10.1093/nar/gkag478

**Published:** 2026-05-19

**Authors:** Yiling Zhou, Sen Yang, Xiaoli Wang, Yuanhang He, Yao Tian, Jiacai Yi, Yikun Wang, Youchao Deng, Dejun Jiang, Dongsheng Cao

**Affiliations:** Xiangya School of Pharmaceutical Sciences, Central South University, Changsha, Hunan 410013, China; Xiangya School of Pharmaceutical Sciences, Central South University, Changsha, Hunan 410013, China; Xiangya School of Pharmaceutical Sciences, Central South University, Changsha, Hunan 410013, China; Xiangya School of Pharmaceutical Sciences, Central South University, Changsha, Hunan 410013, China; Xiangya School of Pharmaceutical Sciences, Central South University, Changsha, Hunan 410013, China; School of Chinese Medicine, Hong Kong Baptist University, Hong Kong, SAR 999077, China; Xiangya School of Pharmaceutical Sciences, Central South University, Changsha, Hunan 410013, China; Xiangya School of Pharmaceutical Sciences, Central South University, Changsha, Hunan 410013, China; Xiangya School of Pharmaceutical Sciences, Central South University, Changsha, Hunan 410013, China; Xiangya School of Pharmaceutical Sciences, Central South University, Changsha, Hunan 410013, China

## Abstract

CYP450 (cytochrome P450)-mediated drug metabolism is a critical determinant of pharmacokinetics and clinical safety, making comprehensive metabolic profiling essential for rational drug discovery. Here, we present DeepCYP (https://deepcyp.scbdd.com), a freely accessible deep-learning web server for end-to-end CYP450 metabolic profiling. Trained on an expanded dataset and a mechanism-based reaction rule library, DeepCYP uses a multi-task graph neural network (GNN) combined with multi-scale descriptors. Operating directly on 2D molecular graphs, this architecture bridges the entire “pathway-site-product” continuum across nine major CYP isoforms (CYP1A2, CYP2A6, CYP2B6, CYP2C8, CYP2C9, CYP2C19, CYP2D6, CYP2E1, and CYP3A4) within a unified pipeline. Benchmarking demonstrates that DeepCYP outperforms established tools, including FAME3, SMARTCyp, and BioTransformer 3.0, improving Top-1 and Top-2 ranking metrics by over 10%. Furthermore, the server supports high-throughput batch processing, capable of evaluating ~280 molecules per minute. DeepCYP also enhances interpretability through an interactive visualization interface, featuring susceptibility radar charts and dynamic transformation tables. By translating abstract predictions into biological insights, DeepCYP provides a practical tool to accelerate lead optimization and mitigate toxicity risks.

## Graphical Abstract

**Graphical Abstract:**
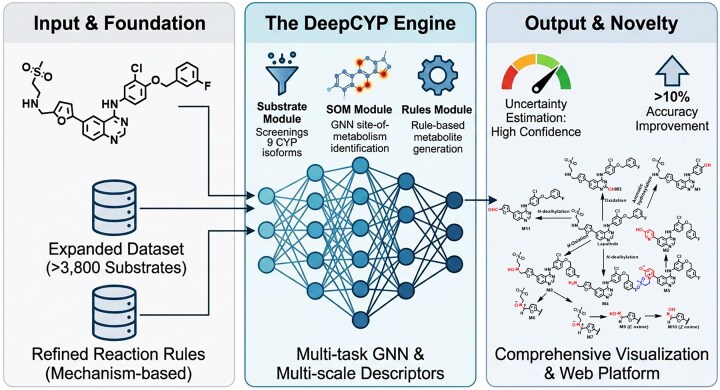

## Introduction

Drug metabolism, also referred to as biotransformation, is a biochemical modification process of xenobiotics mediated by specialized enzyme systems, occurring predominantly within hepatic microsomes [1]. Within this complex human metabolic network, the cytochrome P450 (CYP450) system serves as the primary metabolic enzyme response for Phase I metabolism, governing ~75% of clinical drug transformations [2]. Reflecting its catalytic functions, this enzyme superfamily comprises 18 families and 43 subfamilies, with isoenzymes from the CYP1, CYP2, and CYP3 families accounting for the predominant metabolic activities. As prototypical monooxygenases, CYP450s facilitate the introduction of polar functional groups into substrates via oxidation, reduction, and hydrolysis, thereby streamlining subsequent Phase II conjugation [3]. A drug’s metabolic profile is linked to its pharmacokinetic performance and clinical safety. While metabolic conversion dictates bioavailability and clearance, CYP450-mediated bioactivation can inadvertently generate reactive intermediates or toxic metabolites. Notably, metabolism-induced hepatotoxicity remains a primary driver of drug attrition or withdrawal, accounting for 15%–30% of such cases [4]. A prominent historical example is the global withdrawal of troglitazone in 1997, precipitated by life-threatening liver failure induced by its CYP3A4-generated quinone metabolites [5]. This underscores the profound impact of CYP450 metabolic liabilities on drug candidate viability, making the early and comprehensive elucidation of metabolic pathways an essential prerequisite for drug safety.

To facilitate early-stage metabolic profiling of new molecular entities, high-throughput *in vitro* and *in vivo* screenings have generated extensive experimental datasets [6]. However, the inherent limitations of these empirical methodologies, such as prolonged timelines, prohibitive costs, and ethical constraints restrict screening throughput, catalyzing a paradigm shift toward *in silico* predictive approaches [7]. A diverse array of computational tools has subsequently emerged [8–12], ranging from specialized predictors such as FAME 3 [13], SMARTCyp [14], and BioTransformer 3.0 [15] to comprehensive ADMET platforms [16, 17] and commercial software like StarDrop [18] and MetaSite [19]. Notwithstanding their utility, contemporary platforms remain hindered by two algorithmic and structural bottlenecks. First, a fragmented predictive paradigm persists. Most tools isolate single-dimensional endpoints (e.g. predicting solely the metabolic site or only the downstream product) rather than modeling a holistic representation that correlates the entire “pathway-site-product” continuum. Second, the mechanistic interpretability of results is frequently hindered by suboptimal visualization; most existing models rely on static tabular outputs, which fail to elucidate the complex, multi-step nature of metabolic cascades.

To address current limitations in computational metabolism, we present DeepCYP (https://deepcyp.scbdd.com), a deep learning web server designed for CYP450 metabolic profiling. To meet the needs of contemporary drug discovery, DeepCYP incorporates both algorithmic and data-level improvements. Specifically, it utilizes a dataset that is 30% larger than existing benchmarks (EBoMD [9]) and a reaction rule library expanded by 25%. At its core, the predictive engine utilizes a multi-task graph neural network (GNN) enhanced by multi-scale descriptors. By bypassing complex calculated descriptors, the model generates outputs more rapidly. Furthermore, DeepCYP integrates an uncertainty estimation module and an interactive visualization interface. By quantifying prediction confidence and mapping the reactant-to-product cascade, DeepCYP translates raw numerical scores into biologically interpretable insights. Collectively, these enhancements establish DeepCYP as a practical resource for metabolism-driven rational drug design.

## Method and web server description

### Comprehensive data curation and hierarchical rules encoding

Although the human CYP superfamily contains over 50 enzymes [20], xenobiotic metabolism is predominantly driven by a specific group of hepatic microsomal enzymes rather than mitochondrial forms [21]. For this platform, we focused on nine major microsomal isoforms (CYP1A2, CYP2A6, CYP2B6, CYP2C8, CYP2C9, CYP2C19, CYP2D6, CYP2E1, and CYP3A4). These specific isoforms were selected because they collectively mediate the metabolism of over 90% of clinically approved drugs [22] and represent the primary targets recommended by regulatory agencies for evaluating pharmacokinetics and drug–drug interactions [23].

To construct a robust dataset for metabolic prediction, we integrated data from recent peer-reviewed literature [24–26] like CyProduct [9] and established databases such as DrugBank [27] and BRENDA [28]. All collected molecular structures were preprocessed to exclude complex mixtures and organometallics, neutralize salts, and canonicalize topological features. The final data consist of three components. (i) Data for Pathway Identification (Pathway module): we constructed an enzyme–substrate mapping dataset comprising over 3800 compounds (Supplementary Table S1). The substrate specificity for each compound across the nine major CYP isoforms is encoded as a 9-dimensional binary vector. In this method, if a molecule is metabolized by specific isoforms (e.g. CYP1A2 and CYP3A4), the corresponding positions in the vector are assigned a value of 1, while the non-metabolizing isoforms are labeled as 0. (ii) Data for Site of Metabolism (SoM module): for the SoM prediction task, we compiled a dataset of over 900 annotated molecules. The specific metabolic sites were encoded into atom- and bond-level binary vectors, which served as the target labels for training the GNN. Specifically, atoms or bonds undergoing metabolic changes were labeled as 1, while unaffected ones were labeled as 0. (iii) Data for Metabolite Generation (Rules module): to generate the final metabolite structures, known metabolic transformations were curated and organized into a 15-tier hierarchical library of reaction templates. These templates were manually encoded as SMIRKS patterns [29]. In practice, once the GNN identifies the metabolic pathway and the SoM, the corresponding SMIRKS pattern is applied to that site. This pattern executes the exact structural modifications, such as atom replacement or bond cleavage, to generate the 2D structure of the predicted product.

### Algorithmic foundation and the end-to-end predictive workflow

**Figure 1.**
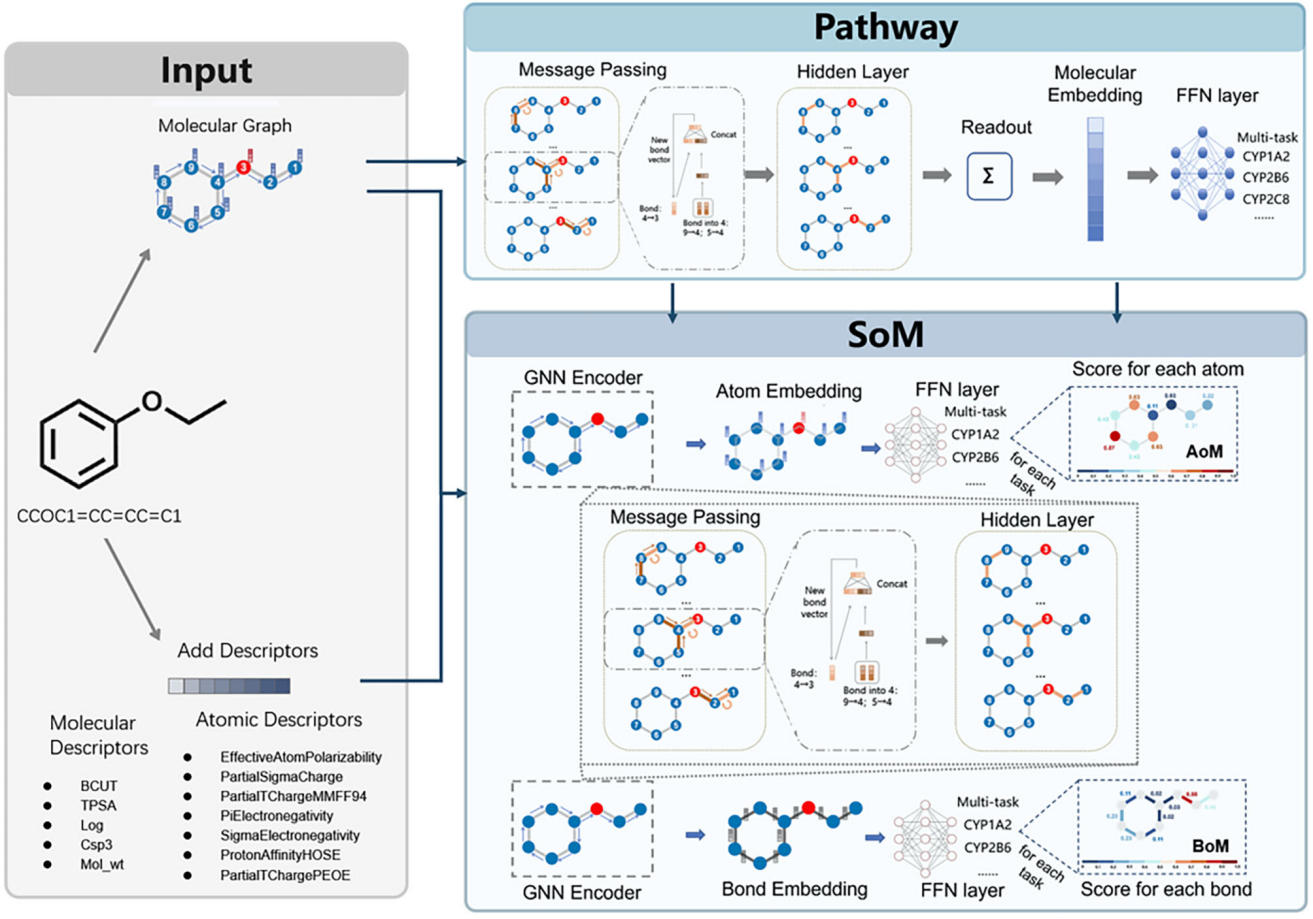
The algorithmic foundation of DeepCYP. The Pathway module uses global GNN embeddings to predict CYP isozyme targets. While the SoM module integrates additional descriptors with local GNN outputs to compute Atom of Metabolism (AoM) and Bond of Metabolism (BoM) probabilities.

The predictive engine of DeepCYP is powered by the DeepMetab framework [30], which comprises two deep learning modules and one rule-based module: a pathway prediction module to identify involved CYP isoforms, a SoM module to locate specific reaction sites, and a rule-based module to generate the final metabolite structures. To compute predictions at a chemical level, the two deep learning modules utilize GNN (Supplementary GNN Framework). Molecules are represented as 2D graphs where atoms function as nodes and bonds as edges. The GNN performs an iterative message-passing process, aggregating local chemical features to capture the distinct topological and electronic microenvironment of each atom and bond [31].The process is detailed in Fig. 1.

For pathway identification, these atomic and bond features are pooled into a global molecular embedding. Here, the deployment of a multi-task GNN architecture allows for the joint optimization of interconnected metabolic endpoints. This facilitates cross-task knowledge transfer, utilizing features learned from data-rich isoforms (e.g. CYP3A4) to enhance predictions for data-scarce targets (e.g. CYP2B6).

Building upon the same multi-task GNN architecture, the SoM module evaluates node and edge representations to compute site-specific reaction probabilities. While pathway prediction is a molecular-level task where GNN-derived basic features are generally sufficient, SoM identification requires pinpointing exact atomic reaction centers. Because GNN captures the local chemical environment through basic node and edge features, these standard embeddings do not fully quantify the electronic densities and steric effects governing reactivity [32]. To address this, the SoM module employs a multi-scale representation strategy. It fuses GNN structural embeddings with atomic-level reactivity descriptors and global molecular properties. This fusion provides the additional physical grounding required to distinguish chemically similar atoms and bonds for mechanistic modeling.

Crucially, while the model weights are optimized using training sets, the predictions are not merely database lookups. By capturing structural correlations, such as inductive and resonance effects [33, 34], the multi-task GNN learns latent catalytic patterns that align with established biochemical principles [35–37]. This enables the platform to generalize and compute metabolic probabilities for completely novel chemical structures.

To translate these SoM predictions into chemical structures, the rule-based metabolite generation module utilizes reaction templates encoded as SMIRKS patterns, which are substructure-centric rather than molecule-specific. By defining documented biotransformations as localized atomic changes and bond modifications (or cleavages), these rules operate independently of the overall molecular scaffold. Consequently, once the GNN identifies a reactive site on a new chemotype, the Rules module applies the corresponding structural modification, enabling the generation of right metabolites for structurally novel compounds.

**Figure 2.**
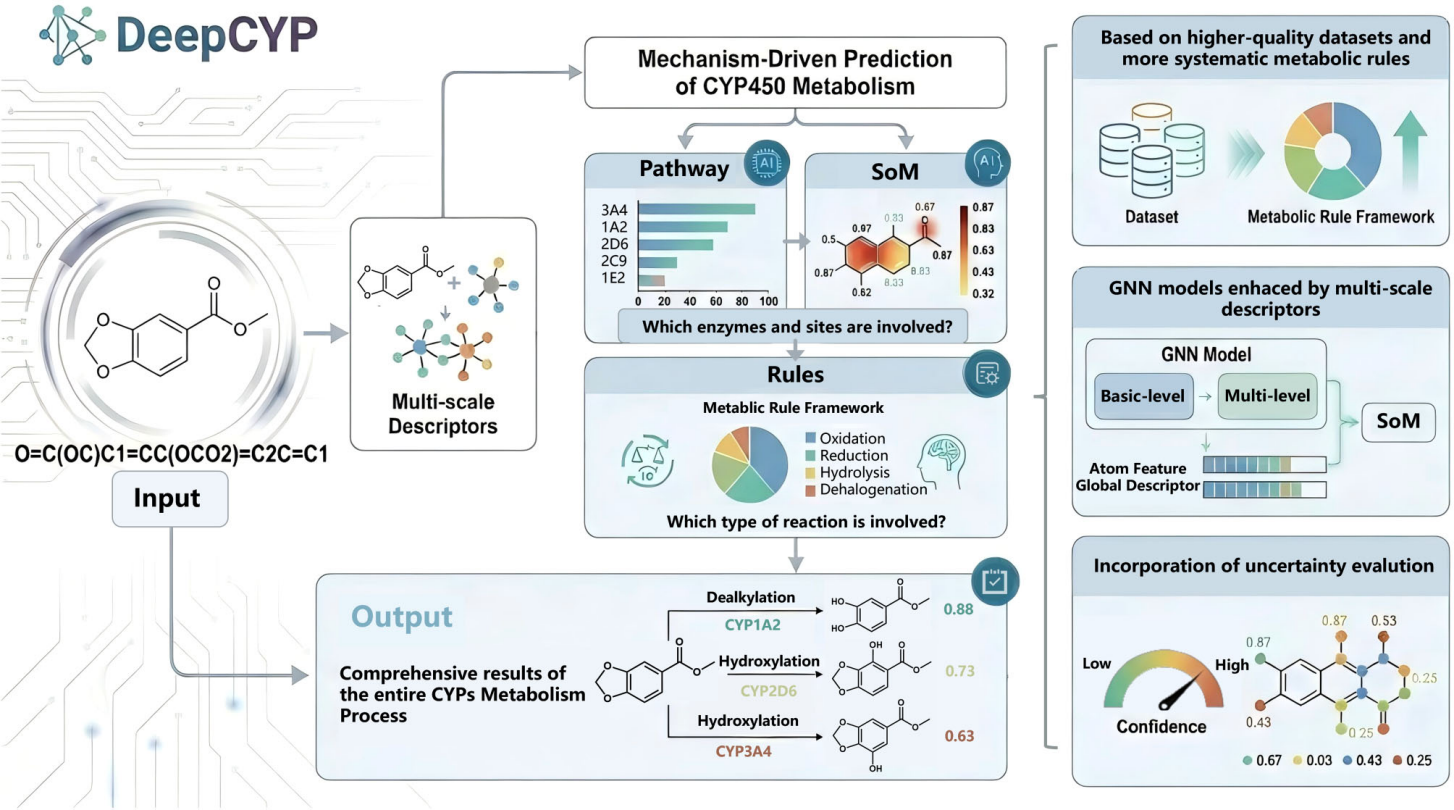
The overall architecture and comprehensive workflow of the DeepCYP platform. The end-to-end pipeline processes molecular inputs (SMILES) through a three-stage cascade: (i) pathway/isoforms identification; (ii) SoM pinpointing; and (iii) rule-based metabolite generation. Key technical advancements (right panel) include: (i) expanded high-quality datasets and refined metabolic rules; (ii) multi-task GNN enhanced by multi-scale descriptors; and (iii) an uncertainty estimation module providing confidence labels to guide experimental validation.

The complete workflow of the DeepCYP platform, illustrated in Fig. 2, comprises five functional modules to sequentially predict metabolic pathways, sites, and products. The process begins with the input module, which standardizes molecular structures into model-ready graph representations. These tensors are processed by the Pathway module to identify the involved CYP450 isoforms among the nine major enzymes. Based on these pathway predictions, the SoM module locates the specific reaction sites. Additionally, a built-in uncertainty estimation module (Supplementary Fig. S1 and Supplementary Table S3) evaluates these predictions to generate quantified confidence labels, ensuring the reliability of the results. The identified sites are then routed to the Rules module, where transformation templates are applied to generate the corresponding metabolite structures. Finally, the output module compiles these results into an interactive visualization, displaying the complete predicted metabolic profile.

### Rigorous model validation and evaluation framework

To ensure the performance and robustness of DeepCYP, we employed a five-fold cross-validation strategy. Ablation studies were conducted on both the Pathway and SoM modules to quantify the contributions of the multi-task learning architecture and the multi-scale descriptors. Model optimization was driven by the Adam optimizer [38], with hyperparameters selected via grid search (Supplementary Table S2). To mitigate the impact of random initialization and assess training stability, each training process was independently repeated 10 times, with the top-performing models ultimately selected for web server deployment.

The generalizability of the optimized models was subsequently evaluated on an independent test set and benchmarked against other tools. Recognizing the distinct biochemical nature of our three predictive tasks, we adopted different evaluation methods: (i) Pathway module: the predictive capacity was primarily assessed using the area under the receiver operating characteristic curve (AUC) and accuracy (ACC). To account for class imbalances in experimental metabolic data, these were supplemented with the precision-recall curve (PRC), sensitivity (SEN), and specificity (SPEC) [39]. (ii) SoM module: site identification was evaluated from both a collective cross-dataset (A) and a per-molecule (R) perspective [40]. We utilized AUC, PRC, the Jaccard index, and Top-N ranking metrics to measure site localization and relative ranking precision within molecules. (iii) Rules module: to evaluate the rule-guided product generation, this module similarly incorporated Jaccard and Top-N metrics to assess the validity and accuracy of the generated structures.

### Web server implementation and architecture

To translate our model into an accessible, high-throughput utility, DeepCYP is built upon a multi-tiered web architecture. The backend is developed using the Django framework, integrated with an asynchronous task-queue and caching mechanism. By leveraging a database to temporarily store intermediate molecular calculations, the platform avoids redundant computations and optimizes overall server responsiveness, ensuring the execution of large batch screening tasks.

At the core of this infrastructure lies the advanced predictive engine, powered by the PyTorch framework and the RDKit cheminformatics suite [36]. This engine manages the simultaneous execution of the multi-task GNN architecture and the mechanism-based metabolite generation. Operating directly on 2D molecular graphs without the need for computationally heavy molecular descriptors, this setup achieves good computational efficiency. Furthermore, the frontend is constructed utilizing Bootstrap and JavaScript to provide interactive user experience. Rather than rendering conventional static tables, the frontend translates the “pathway-site-product” predictions and quantified uncertainty metrics into intuitive visualizations.

## Key features

### Expanded high-quality datasets and refined mechanism-based reaction rules

**Figure 3.**
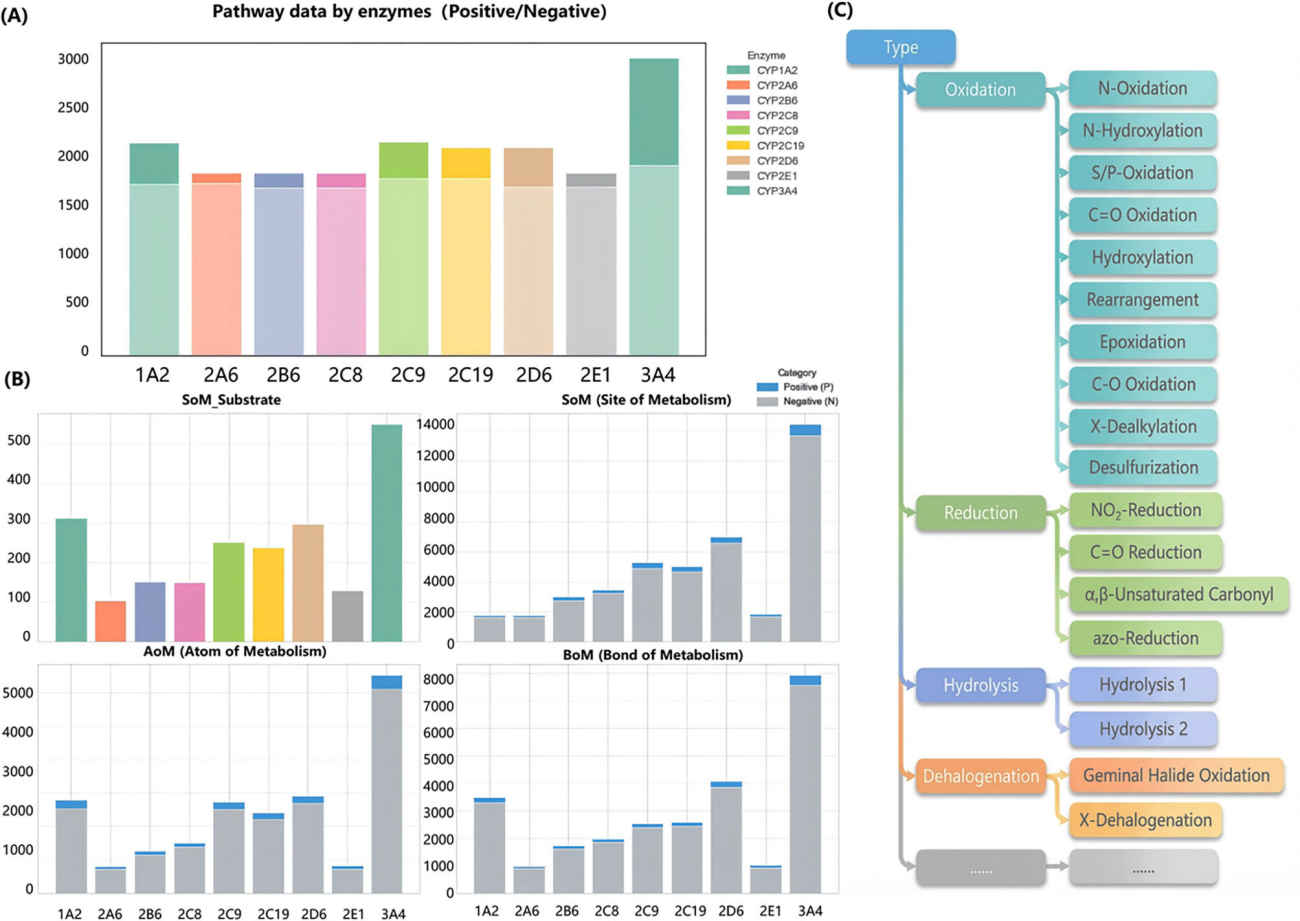
Overview of the expanded datasets and the refined mechanism-based reaction rule library in DeepCYP. (**A**) Pathway data distribution across nine major CYP450 isoforms, showing positive and negative sample proportions. The *y*-axis represents the number of molecules. (**B**) Statistics of the SoM dataset, including total annotated substrates (top-left) and positive/negative distributions for overall SoM (top-right), AoM (bottom-left), and BoM (bottom-right). The *x*-axis represents the specific CYP450 isoforms, and the *y*-axis displays the molecule count. (**C**) Hierarchical reaction rule framework, organized into 4 primary catalytic categories and 15 specific subcategories to guide metabolite generation.

DeepCYP is trained on an expanded experimental dataset covering nine major CYP450 isoforms (CYP1A2, CYP2A6, CYP2B6, CYP2C8, CYP2C9, CYP2C19, CYP2D6, CYP2E1, and CYP3A4), with specific annotations for different modeling tasks (Supplementary Table S1). By curating data from peer-reviewed literature and established databases (e.g. DrugBank and CypReact [41]), we constructed a pathway dataset comprising over 3800 compounds (Fig. 3A) and a SoM dataset of over 900 substrates with AoM and BoM annotations (Fig. 3B). Although this dataset inevitably suffered from class imbalances and uneven isoform distributions, we mitigated these biases during modeling through class-weighting strategies and a multi-task learning architecture. Overall, this represents a >30% increase in data volume compared to previous benchmarks like EBoMD [9], providing a broader basis for model training.

Beyond dataset expansion, DeepCYP mitigates the site-matching ambiguities and misclassification of multi-site biotransformations common in rule-based generators like GLORYx [42] and BioTransformer 3.0 [15]. To achieve this, we developed a combined labeling methodology using both AoM and BoM. Specifically, single-atom reactions (e.g. hydroxylation) are annotated with AoM, while reactions involving atomic pairs or bonds (e.g. dealkylation and epoxidation) are annotated with BoM. This distinction prevents reaction misclassification; for instance, it effectively differentiates N-dealkylation (BoM) from C-hydroxylation (AoM) at the adjacent carbon, resolving a frequent error in GLORYx [42]. Furthermore, we implemented a minimal labeling principle for multi-site reactions. By minimizing redundant site markers, this approach effectively prevents the misidentification of adjacent functional groups as false metabolic sites, a known limitation in BioTransformer 3.0 [15]. Computationally, these AoM and BoM labels are encoded as binary vectors corresponding to the indices of respective atoms or bonds.

Based on this labeling framework, we compiled a library of mechanism-based reaction rules executed via RDKit [43]. These rules are hierarchically classified into 4 primary categories (oxidation, reduction, hydrolysis, and dehalogenation) and 15 secondary subcategories. Crucially, this library incorporates essential metabolic reaction patterns that are frequently absent in existing tools like BioTransformer 3.0 [15]. A notable example of this is dehalogenation (Fig. 3C), a pivotal mechanism underlying the formation of reactive and toxic metabolites.

### Superior predictive performance driven by multi-task GNN

**Figure 4.**
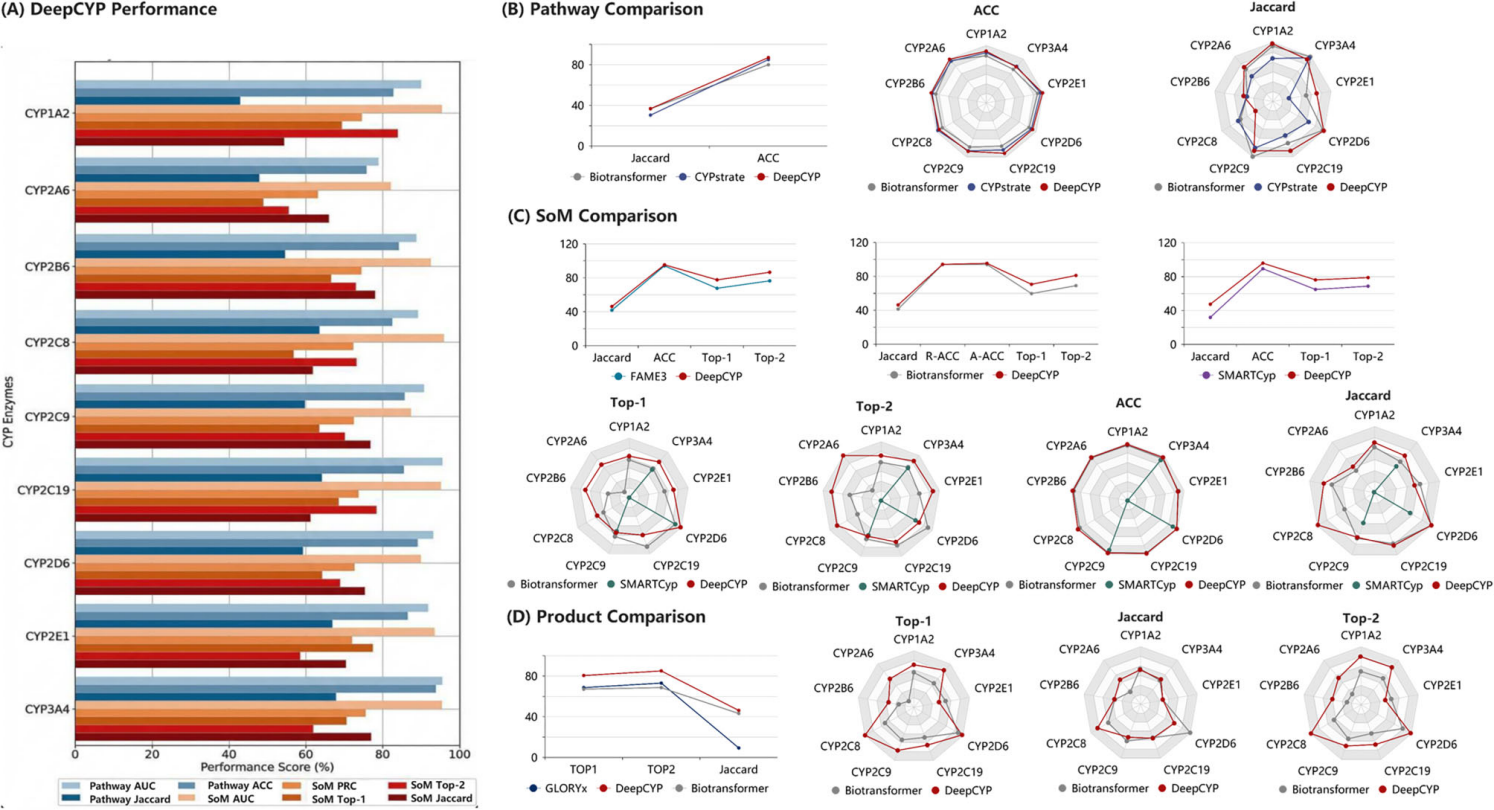
Performance evaluation and benchmarking of the DeepCYP platform. (**A**) DeepCYP performance: predictive metrics (AUC, ACC, Jaccard, Top-1, and Top-2) for pathway and SoM identification across nine CYP isoforms. Right panels: comparative benchmarking against state-of-the-art tools across the three-stage metabolic pipeline; (**B**) pathway identification versus BioTransformer and CYPstrate; (**C**) SoM prediction versus FAME 3, BioTransformer 3.0, and SMARTCyp; and (**D**) product generation versus GLORYx and BioTransformer 3.0.

The algorithmic core of DeepCYP lies a multi-task GNN architecture, enhanced by multi-scale local and global descriptors. Five-fold cross-validation and further comparison confirm that this framework achieves superior predictive performance across the entire “pathway-site-product” continuum (Supplementary Tables S4 and S5, and Fig. 4).

For pathway identification, DeepCYP achieved average metrics of 88.84% AUC, 50.51% AUPRC, and 82.42% ACC across nine isoforms. Notably, while the model performed optimally on data-rich isoforms such as CYP3A4 (AUC of 94%), it also effectively mitigates the impact of data scarcity. By leveraging the multi-task architecture to facilitate cross-task knowledge transfer, DeepCYP utilized representations from data-rich isoforms to improve predictive accuracy for data-scarce targets (e.g. CYP2A6 or CYP2B6), outperforming single-task baseline models. This robustness was even more pronounced in SoM prediction, where the model reached an average AUC of 94.45%, an average Top-2 of 84.75%, and an average Jaccard of 46.17%. Crucially, even for data-limited enzymes, the SoM AUC consistently exceeded 91%. Benchmarking results further revealed that the multi-task approach provided an average accuracy gain of ~4% over traditional single-task models, validating the efficacy of shared architectural representations in capturing common catalytic features across the CYP450 superfamily.

Furthermore, the integration of multi-scale descriptors, particularly atom-level quantum mechanics reactivity information improved the model’s generalization and chemical interpretability. The impact of these descriptors was most evident in small-sample enzyme scenarios. For instance, the Top-2 for CYP2A6 increased by over 3%, reaching 91%. Beyond this, across the entire three-stage metabolic pipeline, DeepCYP outperformed established tools (e.g. CYPstrate [44], FAME 3 [13], SMARTCyp [14], GLORYx [42], and BioTransformer [15]) in pathway, SoM, and product-prediction phases, achieving remarkable enhancements exceeding 10% in product Top-1 and Top-2 ranking metrics, particularly for data-limited isoforms. These results indicate that integrating multi-task deep learning with multi-scale descriptors effectively improves the predictive bottlenecks associated with minor isoforms and novel chemical scaffolds.

### Mechanism-driven, end-to-end prediction and comprehensive visualization

Most existing platforms focus on specific metabolic endpoints, for instance, SMARTCyp [14] is limited to predicting only three CYP450 isoforms, widely used tools such as FAME 3 [13] and XenoSite [45] perform SoM prediction without generating downstream metabolite structures. However, DeepCYP introduces an end-to-end predictive framework. The platform covers nine major CYP450 isoforms, responsible for the majority of Phase I drug biotransformations. By integrating pathway identification, SoM prediction, and metabolite structure generation, DeepCYP models the complete “pathway-site-product” continuum.

**Figure 5.**
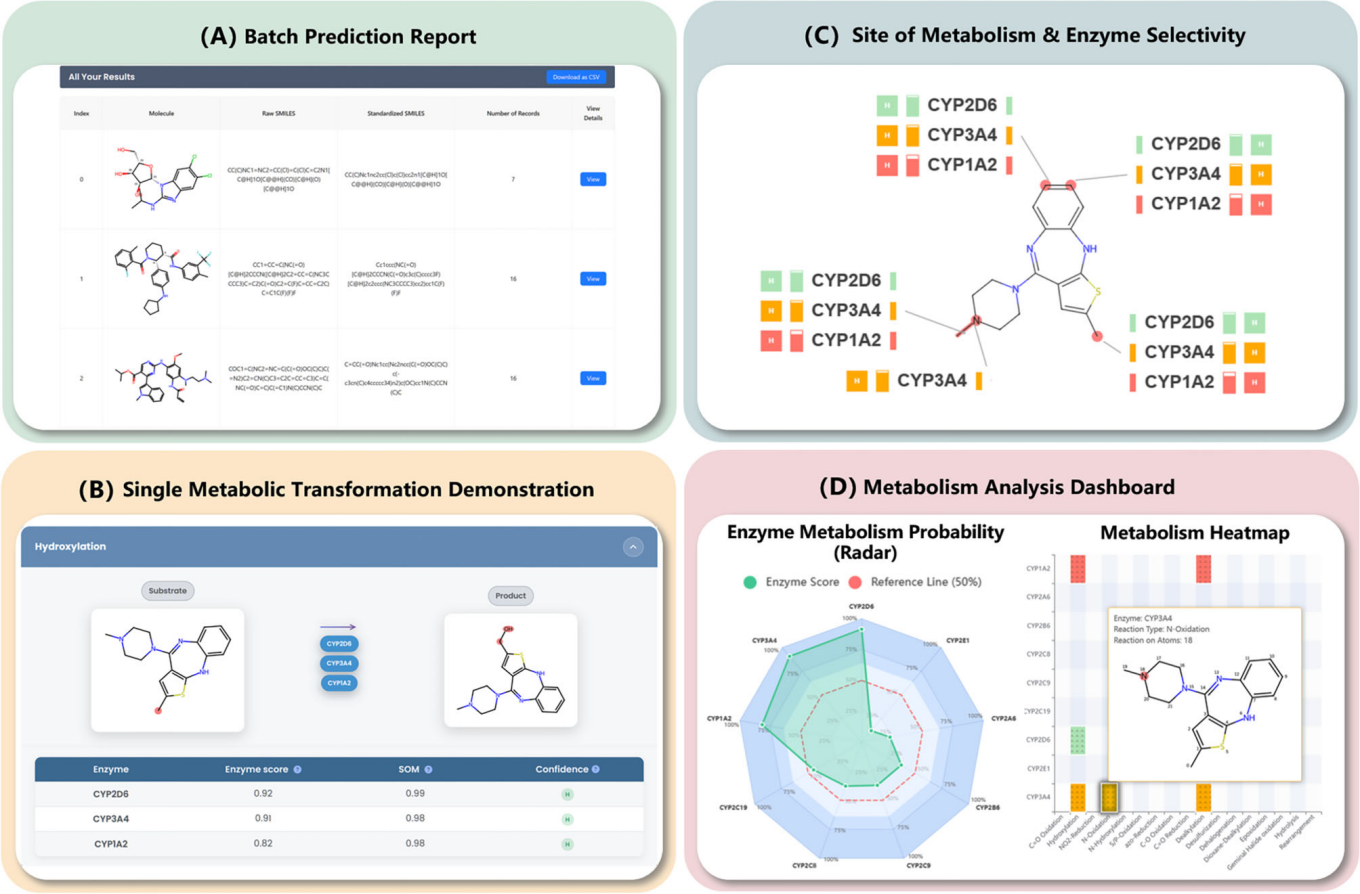
Interactive graphical user interface and visualization modules of the DeepCYP web server. (**A**) Batch prediction report: a concise tabular summary for high-throughput molecular screening. (**B**) Single metabolic transformation: a demonstration of the reactant-to-product conversion, including prediction scores and confidence labels. (**C**) Results visualization: a 2D molecular graph pinpointing predicted SoMs and isoform selectivity. (**D**) Metabolism analysis dashboard: statistical overviews featuring an enzyme susceptibility radar chart and a reaction type heatmap.

To facilitate data interpretation, DeepCYP provides an interactive visualization interface for both single-molecule and batch predictions (Fig. 5). For single-molecule queries, the results feature a 2D molecular graphic that highlights predicted SoMs, annotated with the involved CYP isoforms and confidence scores (Fig. 5C). Clicking a specific reactive site navigates the user to its corresponding detailed transformation entry (Fig. 5B). To provide a metabolic overview, the platform generates analytical charts, including a radar chart summarizing metabolic pathway susceptibility across different CYP isoforms, and a heatmap displaying the statistical relationship between metabolic enzymes and reaction types. Interacting with a specific block in the heatmap highlights the corresponding metabolic sites on the molecular graph (Fig. 5D). Below these summaries, the specific metabolic transformation entries map the reactant-to-product conversion, illustrating the rule-based generation of each metabolite (Fig. 5B). For batch submissions (Fig. 5A), a summary interface enables users to review the overall results before accessing specific detail pages. Finally, to integrate smoothly into practical research workflows, DeepCYP supports the one-click generation of comprehensive PDF reports and the direct downloading of all high-resolution visualization charts, ensuring that users can rapidly document and utilize these insights for lead optimization.

## Case study: navigating the metabolic landscape of olanzapine

To demonstrate the practical utility of DeepCYP in navigating complex biotransformation landscapes, we conducted a representative case study on olanzapine, a second-generation atypical antipsychotic [46]. Olanzapine serves as an ideal benchmark due to its well-characterized and promiscuous metabolic profile, which is primarily orchestrated by multiple CYP450 isoforms in humans, specifically CYP1A2, CYP2D6, and CYP3A4 [47, 48].

**Figure 6.**
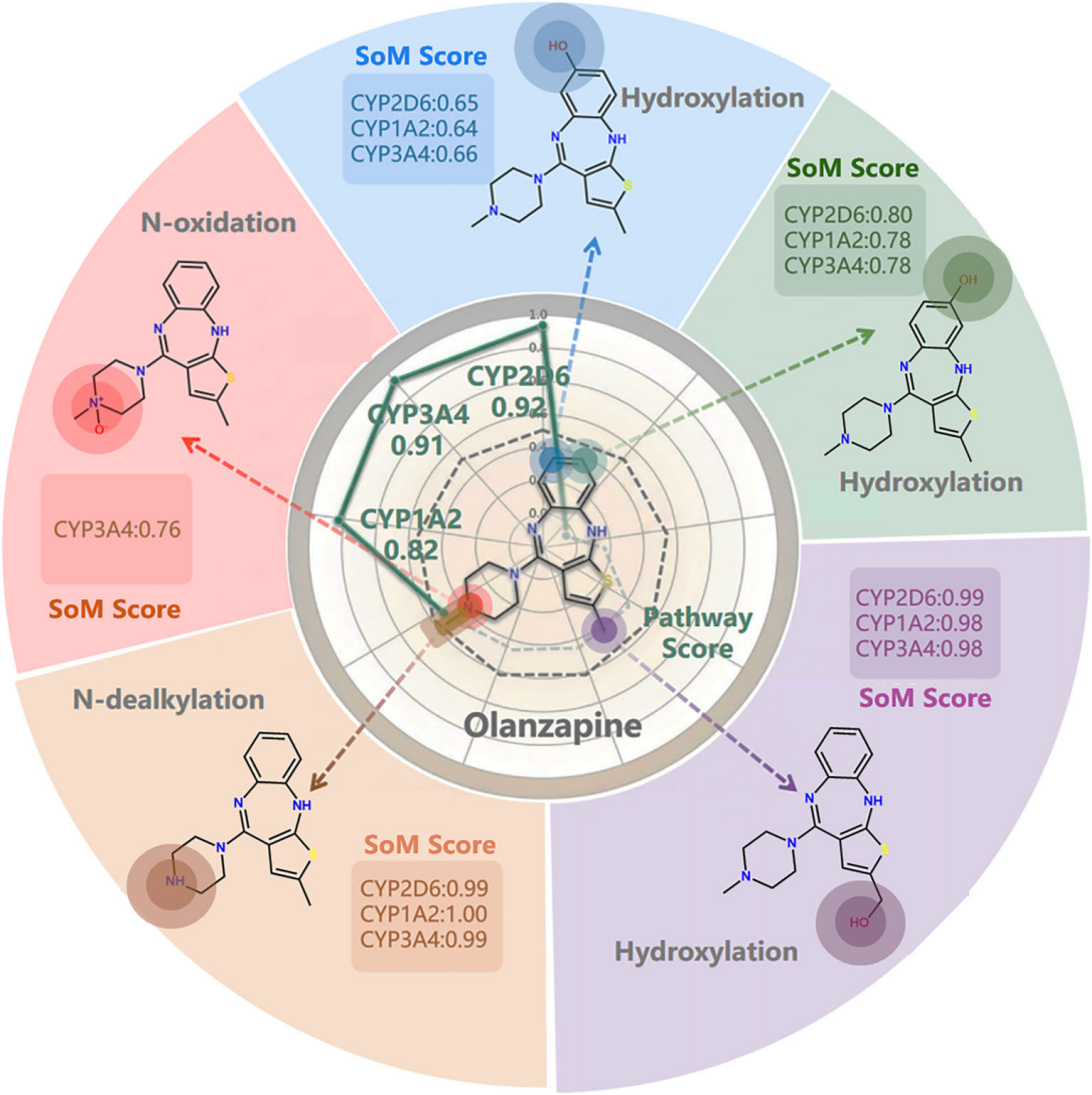
Comprehensive metabolic profiling and the “pathway-site-product” continuum prediction for olanzapine. The central radar chart illustrates CYP isoform susceptibility. Peripheral color-coded sectors display predicted SoMs, biotransformation types (N-oxidation, hydroxylation, and N-dealkylation), and the resulting metabolite structures with their respective isoform-specific prediction scores.

Upon submitting the SMILES string or 2D structure of olanzapine into the DeepCYP web interface, the platform’s multi-task GNN architecture evaluates its susceptibility across all nine major CYP isoforms. As visualized in the chart (Fig. 6), the platform correctly identifies olanzapine as a promiscuous substrate. The built-in uncertainty estimation module assigned high confidence probability scores to CYP2D6 (0.92), CYP1A2 (0.91), and CYP3A4 (0.82), a ranking that aligns with established clinical pharmacokinetics [47, 48].

Moving from pathway identification to site-specific mapping, DeepCYP’s 2D visualization highlights the reactive loci via color-coded spheres. The model successfully pinpointed clinically documented soft spots, offering a mechanistic interpretation that matches known metabolic results: (i) N-dealkylation via CYP1A2: pharmacological studies identify CYP1A2 as the primary engine for the N-dealkylation of olanzapine [49]. Our GNN model accurately captured this atom-level microenvironment, identifying the piperazine N-methyl group as the predominant reactive locus with an exceptional prediction score of 1.00. Subsequent rule-based product generation displayed the formation of 4′-*N*-desmethylolanzapine, a major circulating metabolite. (ii) Multi-site hydroxylation via CYP2D6: clinical data suggest that CYP2D6 (and to a lesser extent CYP3A4) mediates both aromatic and aliphatic hydroxylations [49]. DeepCYP successfully resolved this multi-site ambiguity, accurately predicting the hydroxylation of the 2-methyl group on the thiophene ring (highest predictive confidence of 0.99 for CYP2D6) and the aromatic ring. These loci predictions triggered the generation of 2-hydroxymethylolanzapine and 7-hydroxyolanzapine, respectively. (iii) Minor N-oxidation pathway: the platform flagged the tertiary amine on the piperazine ring as a potential site for N-oxidation, assigning it a moderate predictive score of 0.76 [48]. Rather than discarding this as noise, the platform’s confidence estimation appropriately categorized it as a minor, yet viable, transformation. This is corroborated by human metabolic profiles where secondary metabolites like olanzapine-*N*-oxide consistently appear. The model’s ability to capture this minor route underscores its predictive sensitivity.

## Comparison with state-of-the-art computational tools

To comprehensively evaluate the practical utility of DeepCYP, we benchmarked it against established platforms, including StarDrop [18], FAME 3 [13], SMARTCyp [14], GLORYx [42], and BioTransformer 3.0 [15] (summarized in Table 1). The limitation of existing tools is their isolated endpoints like focusing only on SoM prediction or product generation. In contrast, DeepCYP bridges the “pathway-site-product” continuum within a unified pipeline. Driven by its multi-task GNN architecture and mechanism-based reaction rules, DeepCYP outperforms established baselines, achieving a >10% improvement in critical Top-1 and Top-2 ranking metrics.

**Table 1.** Feature and performance comparison between DeepCYP and existing web-based metabolic prediction tools

Feature/tool	DeepCYP	StarDrop	BioTransformer 3.0	FAME 3	SMARTCyp	GLORYx
Endpoint coverage	Pathway, SoM, product	Pathway, SoM, product	Pathway, product	SoM	SoM	SoM, product
IsoformCoverage	9	None	9	None	3	None
Algorithmic core	Multi-task GNN + rules	Computational chemistry + machine learning	Machine learning+Rule-based	Machine learning	Reactivity rules	Machine learning
Top-2 accuracy	+++++	+++	+++	++++	++	+++
Confidence/ uncertainty	Yes	No	No	No	No	No
Batch processing	Yes (high-speed)	Yes	No	Yes	Yes	Yes
500-molecule runtime	107 s	>12 h	17 280 s	12 204 s	500 s	3000 s
Visualization	Full-pipeline visualization	Full-pipeline visualization	Table	Table	Table	Table
Accessibility	Web server	Paid software	Web server	Web server	Web server	Web server

*Endpoint coverage: prediction across the pipeline (pathway, SoM, and products); isoform coverage: number of supported CYP isoforms (“none” indicates general metabolic prediction); Top-2 accuracy: mean accuracy for CYP-SoM identification on the DeepMetab test set. Legend: +++++ (>85%), ++++ (75%–85%), +++ (65%–75%), ++ (55%–65%); confidence/uncertainty: availability of reliability scores or uncertainty quantification; batch processing/runtime: high-throughput capability; runtime is the average of three 500-molecule trials; visualization/accessibility: graphical representation depth and tool deployment mode.

Beyond predictive accuracy, DeepCYP provides high computational efficiency. This stems from two foundational algorithmic advantages. First, unlike traditional machine learning or QSAR models that rely on the computationally expensive descriptors, our GNN framework operates directly on 2D molecular graphs. Second, the multi-task learning architecture enables the simultaneous evaluation of multiple CYP isoforms and metabolic endpoints within a single forward pass, eliminating the redundant feature extraction required by ensembles of single-task models. Consequently, in our batch-processing evaluations, DeepCYP executed metabolic profiling for 500 molecules in 107 s. Furthermore, recognizing the inherent risks of relying on “black-box” AI models, DeepCYP integrates an interactive visualization interface with an uncertainty estimation module, annotating each prediction with confidence labels to guide experimental validation. Ultimately, unlike expensive commercial suites, DeepCYP is deployed as a freely accessible web server, providing a practical tool for metabolic profiling and lead optimization.

## Conclusions and future plans

In this study, we present DeepCYP, a freely accessible web server designed to overcome the fragmented endpoints and limited visualizations capabilities of existing metabolism prediction tools. By integrating a multi-task GNN with multi-scale descriptors, DeepCYP completes “pathway-site-product” continuum of CYP450-mediated biotransformations within a single forward pass.

Looking forward, future updates to the DeepCYP platform will focus on expanding its biological scope and computational accessibility. While the current system covers CYP450-mediated metabolism, we aim to extend the multi-task learning framework to include Phase II conjugation enzymes (e.g. UGTs and SULTs) and other non-CYP oxidoreductases. This extension will enable the simulation of broader metabolic clearance pathways. To support the computational chemistry community, we will also plan to develop an application programming interface to integrate the predictive engine into external drug discovery pipelines. Furthermore, we plan to refine the visualization capabilities by implementing interactive network representations and improving batch analysis reports for detailed data interpretation.

## Supplementary Material

gkag478_Supplemental_File

## Data Availability

DeepCYP is publicly accessible without registration at https://deepcyp.scbdd.com. Results are promptly displayed on the website and available for download in optional formats.
