# Supplementary material for: DeepCYP: an integrated deep learning web server for the holistic “pathway-site product” prediction of CYP450 metabolism
Source: Nucleic Acids Res. 2026 May 19;54(W1):W22–32. doi: 10.1093/nar/gkag478 (PMC13355065; doi:10.1093/nar/gkag478)
Supplement: gkag478_Supplemental_File [file gkag478_supplemental_file.pdf]

Supplementary Information for

'DeepCYP: an integrated deep learning web server for the holistic "pathway-site-product" prediction of CYP450 metabolism'

Yiling Zhou[1, †], Sen Yang[1, †], Xiaoli Wang[1, †], Yuanhang He[1], Yao Tian[1], Jiacai Yi[2], Yikun Wang[1,*], Youchao Deng[1, *], Dejun Jiang[1, *], Dongsheng Cao[1, *]

[1]Xiangya School of Pharmaceutical Sciences, Central South University, Changsha 410013, Hunan, P.R. China
[2]School of Chinese Medicine, Hong Kong Baptist University, Hong Kong SAR 999077, China

[†] These authors contributed equally to this work.
*Corresponding authors

Corresponding authors
Dongsheng Cao
Email: oriental-cds@163.com

Table of Contents

GNN Framework ... 3

Uncertainty estimation method ... 4

Supplementary Figure 1. Schematic Representation of Uncertainty (Confidence) Estimation in DeepCYP ... 5

Supplementary Table 1. Curated training and test datasets for 9 cytochrome CYP enzymes ... 5

Supplementary Table 2.The hyperparameters for multi-task prediction models ... 6

Supplementary Table 3. The uncertainty threshold for CYP450 isoforms ... 6

Supplementary Table 4. Performance assessment of the Pathway prediction model through 5-fold cross-validation ... 7

Supplementary Table 5. Performance assessment of the SoM prediction model through 5-fold cross-validation ... 8

REFERENCES ... 13

GNN Framework

The core computational engine of our predictive platform is built upon a highly customized Graph Neural Network (GNN) architecture, a deep learning paradigm intrinsically designed to map non-Euclidean molecular topologies into high-dimensional latent spaces.(1) To address distinct metabolic endpoints, this framework is structurally bifurcated into two specialized modules. The Pathway Module evaluates the macroscopic metabolic susceptibility of an entire molecule against specific enzymes, whereas the Site of Metabolism (SoM) Prediction Module pinpoints the precise microscopic reactive loci, such as atoms or bonds, within the molecular structure. The execution of this GNN framework entails a sequential workflow comprising feature initialization, iterative message passing, and final representation readout.

Unlike traditional node-centric GNN, our algorithm commences by initializing directed edge representations to accurately capture localized chemical environments.(2) The initial state of a directed edge from atom v to atom w is constructed by fusing the intrinsic properties of the bond with the features of its source atom. Importantly, to prevent the redundant tottering of information, this initial message strictly enforces directionality. The initialization prior to the first message-passing step is mathematically defined as $\mathrm{h}_{\mathrm{vw}}^{(0)} = \mathrm{ReLU}(\mathrm{W}_{\mathrm{init}}[\mathrm{x}_{\mathrm{v}}||\mathrm{e}_{\mathrm{vw}}])$, where $\mathrm{W}_{\mathrm{init}}$ denotes a learnable weight matrix, $[\mathrm{x}_{\mathrm{v}}||\mathrm{e}_{\mathrm{vw}}]$ represents the concatenation of the initial node features $(\mathrm{x}_{\mathrm{v}})$ for atom v and the bond features $(\mathrm{e}_{\mathrm{vw}})$ for bond vw, and ReLU serves as the non-linear activation function.

During the subsequent message-passing phase, the network captures higher-order topological interactions by propagating information across the molecular graph. Rather than updating node hidden states directly, our architecture systematically refines the directed edge hidden states $(\mathrm{h}_{\mathrm{vw}})$ and messages $(\mathrm{m}_{\mathrm{vw}})$. At each iteration t, the message is aggregated from adjacent connected bonds, deliberately excluding the immediate reverse pathway to ensure robust structural learning. This aggregation is expressed as $\mathrm{m}_{\mathrm{vw}}^{(\mathrm{t}+1)} = \sum_{\mathrm{k}\in\mathrm{N}(\mathrm{v})/\{\mathrm{w}\}} \mathrm{h}_{\mathrm{kv}}^{(\mathrm{t})}$, which is then utilized to update the hidden state via $\mathrm{h}_{\mathrm{vw}}^{(\mathrm{t}+1)} = \mathrm{ReLU}(\mathrm{W}_{\mathrm{msg}} \cdot \mathrm{m}_{\mathrm{vw}}^{(\mathrm{t}+1)})$. In these equations, $\mathrm{N}(\mathrm{v})\backslash\{\mathrm{w}\}$ denotes the neighboring atoms of v excluding the destination atom w, and $\mathrm{W}_{\mathrm{msg}}$ represents the learned weight matrix with a designated hidden dimension. Following T iterations of this propagation, the ultimate hidden states for both atoms and bonds are derived by aggregating the converged edge features.

The strategy for generating the final embeddings diverges based on the functional objective of the respective module. For the Pathway Module, the updated atomic and bond states are pooled and aggregated to construct a comprehensive, graph-level molecular embedding. Conversely, the SoM Prediction Module circumvents this global aggregation, directly preserving the individualized, high-resolution atomic (node) and bond (edge) embeddings to facilitate granular, site-specific evaluations.(3) Ultimately, these finely-tuned embeddings are channeled into downstream task-specific Feed-Forward Neural Networks (FFNNs). Within the scope of our study, which encompasses nine major CYP450 isoforms, these FFNNs translate the latent embeddings into predictive probabilities. Consequently, the

Pathway module yields a macro-level classification indicating overall enzyme-substrate specificity, whereas the SoM module outputs micro-level probabilities at each node or edge, accurately delineating the metabolic liability of specific functional groups.(4)

Uncertainty estimation method

To rigorously quantify the predictive reliability of our framework and mitigate the inherent overconfidence often observed in deep learning architectures, DeepCYP incorporates an advanced Uncertainty Estimation module. This framework leverages an ensemble learning strategy established during the model training phase. Specifically, utilizing a five-fold cross-validation scheme, we independently generated an ensemble of five distinct predictive models for both the pathway identification and the Site of Metabolism (SoM, including AOM and BOM) tasks. For any given input molecule, the epistemic uncertainty of the prediction is mathematically captured by measuring the variance (σ^2) of the probability scores outputted across these ensemble models.(5) If x_i represents the predicted probability from the i-th model andμdenotes the mean prediction across all N=5 models, the ensemble variance is calculated as$\sigma^2 = \frac{1}{N}\sum_{i=1}^{N}(x_i - \mu)^2$. This variance serves as the foundational metric for model uncertainty, where a higher variance indicates significant algorithmic disagreement, thereby reflecting lower predictive confidence.(6) To translate this continuous variance metric into an intuitive, binary confidence label—categorized as either High-confidence (H) or Low-confidence (L)—we established statistically optimized variance thresholds (θ). A specific prediction is assigned an 'H' label if its ensemble variance satisfies $\sigma^2 < \theta$, and an 'L' label if $\sigma^2 \geq \theta$. The optimal threshold for each distinct predictive task and CYP450 isoform was determined by maximizing Youden's J statistic (J-score) on the validation sets.(7) The J-score is defined as $J = \frac{TP}{TP+FN} + \frac{TN}{TN+FP} - 1$, where TP, FN, TN, and FP represent the counts of true positives, false negatives, true negatives, and false positives, respectively. By maximizing this index, we ensure an optimal equilibrium between sensitivity and specificity when differentiating reliable from unreliable predictions. The exact empirically derived θ values for AOM and BOM across the nine major CYP450 isoforms are comprehensively detailed in **Table S3**.

Crucially, because an authentic in vivo biotransformation necessitates both the successful recognition of the molecule by the metabolizing enzyme and the precise chemical modification at a specific structural locus, the ultimate confidence label provided by DeepCYP is evaluated holistically. Our platform enforces a strict combinatorial logic: the final uncertainty label for a complete metabolic event is designated as High (H) if and only if both the upstream pathway prediction and the corresponding downstream SoM prediction independently achieve an 'H' rating. If either the enzyme-substrate matching or the site identification exhibits high variance (indicating low confidence), the overall transformation is conservatively flagged as Low (L). As visually demonstrated in **Supplementary Figure 1**, this integrated dual-condition assessment enables the platform to accurately annotate predicted molecular structures. Each identified reactive site is

explicitly tagged with a composite label detailing the probability score, reaction type, and integrated confidence level (e.g., <0.92; Dealkylation; H>), empowering medicinal chemists to judiciously prioritize subsequent experimental validations based on quantified computational reliability.

Supplementary Figure 1. Schematic representation of uncertainty (confidence) estimation in DeepCYP

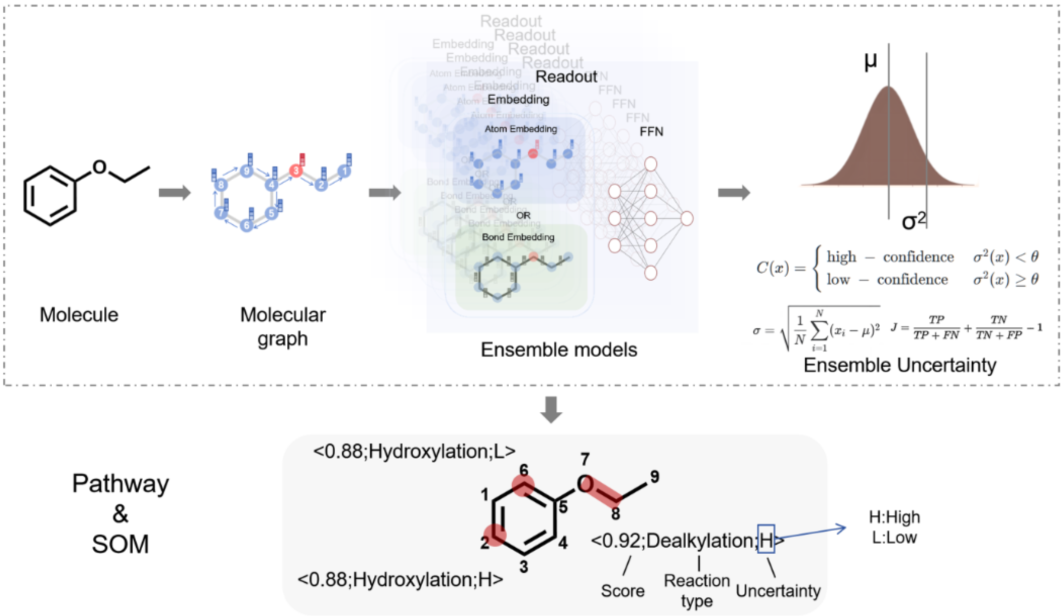

The overall **uncertainty** is classified as **high (H)** only when both the **pathway** and the **SOM** are rated as high

Supplementary Table 1. Curated training and test datasets for 9 cytochrome CYP enzymes

	Enzyme	CYP1A2	CYP2A6	CYP2B6	CYP2C8	CYP2C9	CYP2C19	CYP2D6	CYP2E1	CYP3A4
Training	substrate	416	102	154	157	369	314	396	148	1081
	non-substrate	1719	1736	1684	1681	1779	1776	1694	1690	1910
	Substrates(SoM)	313	104	152	150	251	238	297	129	550
	Aom(SoM)	276	80	125	115	224	210	209	98	420
	non-Aom(SoM)	2515	718	1135	1374	2502	2201	2694	724	6105
	Bom(SoM)	214	72	108	105	152	142	217	90	361
	non-Bom(SoM)	3272	890	1609	1850	2371	2441	3854	923	7561
Test	substrate	40	14	12	12	33	29	27	8	63
	non-substrate	115	120	123	120	114	120	117	122	113
	Substrates(SoM)	17	9	13	14	17	16	27	10	40
	Aom(SoM)	293	86	123	112	220	197	213	107	390
	non-Aom(SoM)	2649	793	1136	1310	2532	2145	2671	798	5713
	Bom(SoM)	230	83	113	108	157	153	225	115	348
	non-Bom(SoM)	3438	1083	1657	1884	2627	2578	3949	1169	7182

Supplementary Table 2. The hyperparameters for multi-task prediction models

Enzyme	Pathway	SoM
Epochs	125	175
Batch size	40	30
Hidden size	400	300
MPN layer	5	3
Dropout rate	0.1	0.1
Learning rate	1e-3	1e-3

Supplementary Table 3. The uncertainty threshold for CYP450 isoforms

Enzyme	1A2	2A6	2B6	2C8	2C9	2C19	2D6	2E1	3A4
AOM	7.0e-04	3.2e-04	7.6e-04	5.5e-04	4.6e-04	6.4e-04	5.0e-04	4.6e-04	6.5e-04
BOM	7.3e-03	7.4e-03	2.7e-02	2.8e-02	4.9e-03	2.7e-02	2.7e-02	6.9e-03	2.7e-02

Supplementary Table 4. Performance assessment of the Pathway prediction model through 5-fold cross-validation

Enzyme	CYP1A2	CYP2A6	CYP2B6	CYP2C8	CYP2C9	CYP2C19	CYP2D6	CYP2E1	CYP3A4	Avg
AUC(Area Under the Curve)										
Single	88.99±0.02	87.39±0.02	86.66±0.07	85.11±0.01	86.44±0.01	89.19±0.00	91.68±0.02	86.53±0.14	94.28±0.00	88.48±0.03
Multi	89.17±0.00	86.57±0.02	86.78±0.02	86.73±0.01	88.34±0.01	88.53±0.01	90.76±0.00	88.59±0.03	94.09±0.00	88.84±0.00
AUPRC(Area Under Precision-Recall Curve)										
Single	56.25±0.38	41.47±0.29	39.07±0.48	31.08±0.11	48.86±0.27	53.79±0.13	69.42±0.32	43.39±0.45	84.80±0.03	52.02±0.27
Multi	55.87±0.07	34.67±0.21	40.98±0.24	33.93±0.05	48.42±0.09	48.42±0.14	62.66±0.13	46.88±0.15	82.74±0.02	50.51±0.03
ACC(Accuracy)										
Single	79.08±0.09	83.50±0.59	79.82±0.34	82.58±0.07	81.35±0.03	80.43±0.08	81.04±0.22	85.28±0.29	87.85±0.02	82.32±0.19
Multi	80.23±0.02	85.38±0.08	81.93±0.05	79.95±0.02	80.02±0.03	80.55±0.04	81.81±0.02	85.64±0.05	86.29±0.01	82.42±0.03
PRE(Precision)										
Single	43.32±0.20	30.86±1.08	30.46±0.60	28.93±0.49	40.88±0.32	39.34±0.19	46.87±0.59	36.03±1.36	75.82±0.14	41.39±0.55
Multi	45.89±0.03	26.18±0.12	31.17±0.11	27.19±0.02	40.06±0.06	40.14±0.10	48.22±0.10	35.91±0.07	70.85±0.03	40.62±0.04
Recall										
Single	84.99±0.51	65.39±3.63	76.59±2.37	66.42±0.49	72.14±0.41	78.32±0.54	84.66±0.57	65.35±0.97	85.52±0.20	75.48±1.07
Multi	80.69±0.09	61.71±0.72	69.35±0.28	72.71±0.16	80.17±0.11	78.35±0.07	81.82±0.16	68.33±0.35	91.55±0.03	76.07±0.10
SPEC(Specificity)										
Single	77.95±0.20	84.85±0.90	80.31±0.63	84.19±0.13	83.02±0.08	80.80±0.15	80.42±0.40	87.21±0.43	88.77±0.05	83.06±0.33
Multi	80.16±0.05	87.03±0.12	83.27±0.07	80.61±0.03	79.97±0.06	80.91±0.07	81.78±0.06	87.33±0.07	84.15±0.01	82.80±0.05
SEN(Sensitivity)										
Single	84.99±0.51	65.39±3.63	76.59±2.37	66.42±0.49	72.14±0.41	78.32±0.54	84.66±0.57	65.35±0.97	85.52±0.20	75.48±1.07
Multi	80.69±0.09	61.71±0.72	69.35±0.28	72.71±0.16	80.17±0.11	78.35±0.07	81.82±0.16	68.33±0.35	91.55±0.03	76.07±0.10
Jaccard										
Single	39.99±0.13	24.37±0.41	26.47±0.14	24.78±0.20	34.90±0.14	35.24±0.11	42.71±0.36	29.37±0.67	67.13±0.17	36.11±0.26
Multi	40.84±0.01	21.79±0.08	26.85±0.08	24.35±0.02	35.91±0.04	35.58±0.06	43.18±0.05	30.13±0.06	66.27±0.02	36.10±0.02

* Single and Multi denote single-task and multi-task Graph Neural Network (GNN) architectures, respectively

Avg represents the unweighted arithmetic mean of the corresponding performance metrics across all nine evaluated CYP450 enzymes, providing a macroscopic measure of overall model robustness.

134 **Supplementary Table 5.** Performance assessment of the SoM prediction model through 5-fold cross-validation

	Enzyme	CYP1A2	CYP2A6	CYP2B6	CYP2C8	CYP2C9	CYP2C19	CYP2D6	CYP2E1	CYP3A4	W*avg
						R-AUC					
Unoptimized	Single(w/o Atom and Mol)	93.50±1.17	90.39±3.63	92.42±1.86	91.19±2.18	93.65±1.84	93.87±1.84	93.57±1.37	87.15±3.21	94.58±0.64	93.08±1.58
	Single (w/o Mol)	93.82±1.32	91.27±2.81	93.61±1.85	92.39±2.30	94.20±1.70	94.64±1.88	93.64±1.41	88.24±3.51	94.67±0.81	93.58±1.62
	Single (w/o Atom)	93.94±1.40	89.91±2.64	93.28±1.81	92.17±2.85	93.75±1.36	94.06±1.49	93.93±1.78	87.86±2.94	94.69±0.73	93.40±1.58
	Single	94.07±1.13	90.93±3.72	93.14±1.81	92.32±2.23	93.93±1.91	94.21±1.78	93.78±1.34	88.18±3.43	94.84±0.65	93.54±1.59
	Multi(w/o Atom and Mol)	94.83±0.94	93.98±2.23	95.55±1.22	94.87±1.20	95.13±1.15	95.42±1.22	95.17±1.09	91.04±2.55	94.08±0.76	94.57±1.17
	Multi (w/o Mol)	94.85±1.15	94.39±2.19	95.61±0.95	95.02±1.25	95.26±1.28	95.49±1.23	95.08±1.08	91.39±2.66	94.18±1.09	94.67±1.28
	Multi (w/o Atom)	94.78±0.99	94.09±2.15	95.53±1.14	95.10±1.04	95.16±1.17	95.23±1.15	95.15±0.92	91.15±2.41	94.29±0.77	94.63±1.12
	Multi	94.81±1.16	93.92±2.15	95.67±1.08	95.32±1.47	95.51±1.41	95.67±1.31	95.28±1.03	91.23±2.46	94.29±1.24	94.76±1.35
Optimized	Single(w/o Atom and Mol)	93.46±1.19	90.98±2.72	93.34±1.90	91.26±2.26	93.65±1.61	93.61±1.73	93.37±1.46	87.23±3.19	94.93±0.71	93.21±1.54
	Single (w/o Mol)	94.01±1.14	91.94±2.29	93.92±1.73	92.07±2.00	94.05±1.71	94.50±1.46	93.64±1.81	88.31±2.76	95.04±0.84	93.70±1.51
	Single (w/o Atom)	94.26±1.40	90.34±2.64	93.74±1.81	91.96±2.85	93.94±1.36	94.01±1.49	93.88±1.78	87.48±2.94	95.03±0.79	93.56±1.59
	Single	94.03±1.23	91.68±2.69	93.67±1.75	91.87±2.41	93.97±1.49	94.35±1.43	93.99±1.42	87.63±2.91	95.11±0.72	93.66±1.47
	Multi(w/o Atom and Mol)	94.88±0.75	94.25±1.64	95.32±1.02	95.36±1.11	95.20±0.93	95.49±1.17	95.12±0.75	91.32±2.07	94.76±0.70	94.81±0.97
	Multi (w/o Mol)	95.08±0.94	94.09±1.71	95.63±0.86	95.20±1.26	95.35±1.17	95.66±1.24	95.27±0.94	91.24±1.92	94.79±0.79	94.90±1.07
	Multi (w/o Atom)	94.98±1.08	93.98±1.62	95.50±0.85	95.32±1.38	95.34±1.08	95.39±1.19	95.24±1.02	91.46±2.60	94.73±1.02	94.84±1.19
	Multi	95.11±0.89	94.30±1.66	95.66±0.93	95.51±1.14	95.30±1.02	95.41±0.96	95.31±0.94	91.26±1.93	94.95±0.68	94.95±0.99
						A-AUC					
Unoptimized	Single(w/o Atom and Mol)	92.70±1.18	91.48±3.63	91.87±1.87	90.84±2.18	93.47±1.89	92.88±1.86	93.25±1.37	88.34±3.26	93.93±0.64	92.69±1.59
	Single (w/o Mol)	93.08±1.34	91.86±2.78	92.91±1.75	91.84±2.34	93.93±1.70	93.81±1.88	93.14±1.41	89.65±3.51	94.00±0.81	93.14±1.62
	Single (w/o Atom)	92.95±1.42	90.93±2.62	92.76±1.91	91.68±2.83	93.59±1.36	93.34±1.49	93.30±1.88	89.42±2.96	94.03±0.71	92.98±1.59
	Single	93.34±1.13	91.82±3.82	92.58±1.81	91.65±2.23	93.64±1.93	93.44±1.79	93.08±1.34	89.27±3.43	94.21±0.65	93.09±1.60
	Multi(w/o Atom and Mol)	94.14±0.95	95.05±2.24	94.70±1.23	94.20±1.20	95.00±1.16	94.45±1.22	94.66±1.09	93.10±2.55	93.33±0.77	94.17±1.17
	Multi (w/o Mol)	94.35±1.15	94.98±2.19	94.86±0.96	94.32±1.25	94.95±1.28	94.32±1.24	94.63±1.09	92.95±2.66	93.31±1.09	94.17±1.29
	Multi (w/o Atom)	94.22±0.99	94.63±2.15	94.65±1.14	94.87±1.05	95.01±1.17	93.99±1.15	94.47±0.93	93.03±2.42	93.85±0.78	94.25±1.13
	Multi	94.15±1.17	95.11±2.15	94.86±1.09	94.44±1.47	95.04±1.41	94.40±1.32	94.74±1.03	93.07±2.47	93.61±1.25	94.28±1.35
Optimized	Single(w/o Atom and Mol)	92.46±1.20	92.37±2.73	93.24±1.90	91.25±2.27	93.75±1.61	92.87±1.74	92.91±1.46	88.56±3.20	94.35±0.71	92.93±1.54
	Single (w/o Mol)	93.06±1.15	92.68±2.30	93.81±1.73	92.22±2.00	94.09±1.72	93.89±1.46	93.24±1.82	89.41±2.77	94.44±0.84	93.40±1.52
	Single (w/o Atom)	93.07±1.41	91.45±2.64	93.48±1.82	91.94±2.85	93.74±1.37	93.36±1.49	93.21±1.79	88.80±2.94	94.53±0.79	93.19±1.59
	Single	93.03±1.24	92.34±2.69	93.59±1.76	91.80±2.42	94.05±1.49	93.61±1.44	93.20±1.43	89.27±2.92	94.68±0.73	93.35±1.48
	Multi(w/o Atom and Mol)	94.12±0.76	94.89±1.64	94.68±1.03	94.89±1.11	94.97±0.94	94.22±1.18	94.49±0.75	92.90±2.08	93.87±0.70	94.27±0.98
	Multi (w/o Mol)	94.41±0.95	94.68±1.71	94.89±0.87	94.93±1.26	95.21±1.18	94.35±1.24	94.56±0.94	93.15±1.93	93.87±0.79	94.39±1.08
	Multi (w/o Atom)	94.22±1.09	94.83±1.63	94.75±0.85	94.66±1.39	95.18±1.08	94.55±1.19	94.73±1.03	93.08±2.61	93.54±1.03	94.29±1.20

	Multi	94.39±0.89	95.08±1.66	95.14±0.93	95.12±1.14	95.08±1.02	94.23±0.96	94.73±0.94	93.13±1.93	94.18±0.68	94.51±0.99
R-PRCAUC											
Unoptimized	Single(w/o Atom and Mol)	68.29±3.03	71.64±4.84	69.31±4.38	65.34±4.80	65.45±2.88	67.88±3.56	70.81±2.78	68.18±4.63	64.58±1.86	67.35±3.15
	Single (w/o Mol)	69.41±2.84	71.45±4.44	69.32±5.39	65.78±4.06	65.94±2.81	68.22±3.59	71.12±3.08	68.68±4.50	65.05±1.75	67.82±3.12
	Single (w/o Atom)	68.81±2.91	70.86±4.76	68.98±4.16	65.99±3.98	65.72±3.27	68.00±3.24	70.72±4.20	68.14±4.32	64.47±1.86	67.41±3.24
	Single	69.21±2.65	71.53±4.40	69.78±4.85	65.51±4.18	65.91±3.50	67.77±3.59	71.02±3.41	69.26±4.14	64.97±1.90	67.75±3.20
	Multi(w/o Atom and Mol)	70.31±2.14	74.88±3.77	72.57±1.94	69.93±3.65	68.40±2.30	69.68±2.18	71.61±4.89	69.81±4.98	64.45±2.46	69.04±2.96
	Multi (w/o Mol)	70.73±2.83	74.08±4.76	73.30±4.23	69.70±3.44	69.06±2.69	70.19±2.84	71.82±2.65	69.47±4.16	65.16±2.94	69.42±3.13
	Multi (w/o Atom)	70.49±3.16	73.93±3.31	72.97±2.81	69.00±3.71	68.45±2.35	69.87±2.39	71.79±2.87	69.80±2.57	65.24±3.14	69.24±2.92
	Multi	70.53±1.46	74.04±2.44	73.20±3.02	69.25±2.99	68.53±3.60	70.26±2.33	71.53±3.56	69.41±3.85	65.55±3.34	69.35±2.96
Optimized	Single(w/o Atom and Mol)	69.03±2.93	72.75±5.84	71.12±2.38	67.71±4.80	65.71±2.88	68.88±3.56	70.82±2.78	69.89±4.63	66.03±1.86	68.40±3.04
	Single (w/o Mol)	69.76±2.84	72.93±4.44	71.41±5.39	67.78±4.06	66.83±2.81	68.44±3.59	71.15±3.08	70.57±4.50	66.09±1.65	68.72±3.10
	Single (w/o Atom)	69.62±2.91	72.58±4.76	70.99±4.16	67.75±3.98	66.30±3.27	68.84±3.24	70.45±4.20	69.84±4.32	66.25±2.49	68.54±3.40
	Single	69.71±2.65	72.92±4.40	71.55±4.85	68.26±4.18	66.55±3.50	68.59±3.59	70.60±3.31	70.55±4.24	66.40±1.77	68.74±3.16
	Multi(w/o Atom and Mol)	70.89±1.14	73.98±1.77	73.94±1.04	70.95±1.65	68.58±1.30	70.29±1.18	71.64±2.79	69.33±3.98	65.77±1.46	69.64±1.70
	Multi (w/o Mol)	71.24±1.46	74.13±2.44	74.07±1.82	70.70±1.99	69.18±1.60	71.12±1.33	72.26±1.56	69.48±3.85	66.28±1.34	70.07±1.70
	Multi (w/o Atom)	71.14±1.17	74.12±1.31	74.48±1.81	70.38±1.72	69.02±2.35	71.13±2.39	72.48±1.17	69.80±2.87	66.29±1.15	70.10±1.63
	Multi	71.19±1.16	74.31±1.31	74.48±1.81	71.19±1.71	69.43±2.35	71.12±2.39	72.35±1.17	69.67±2.87	66.21±1.14	70.17±1.62
A-PRCAUC											
Unoptimized	Single(w/o Atom and Mol)	60.15±3.03	57.05±4.85	57.57±4.38	51.12±4.80	60.62±2.85	60.19±3.56	58.93±2.79	56.25±4.63	52.02±1.87	56.82±3.15
	Single (w/o Mol)	59.70±2.84	58.96±4.45	59.38±5.39	52.68±4.07	61.45±2.82	61.81±3.59	59.83±3.08	57.35±4.51	52.14±1.76	57.57±3.13
	Single (w/o Atom)	60.95±2.92	58.01±4.77	57.30±4.17	50.32±3.99	60.21±3.28	60.11±3.25	59.66±4.21	54.76±4.33	52.93±1.87	57.09±3.25
	Single	61.73±2.66	59.16±4.41	59.15±4.86	51.49±4.19	61.01±3.50	61.10±3.59	60.19±3.42	56.11±4.15	53.83±1.90	58.04±3.21
	Multi(w/o Atom and Mol)	63.71±2.15	67.45±3.78	66.71±1.95	58.68±3.66	64.32±2.31	65.24±2.19	63.84±4.90	64.74±4.98	54.32±2.47	61.70±2.97
	Multi (w/o Mol)	63.36±1.47	68.06±2.45	67.01±3.04	60.54±2.99	64.69±3.61	65.41±2.34	63.52±3.56	64.35±3.86	54.72±3.35	61.92±2.97
	Multi (w/o Atom)	63.75±2.83	66.14±4.77	65.74±4.24	60.58±3.45	64.50±2.69	65.17±2.85	63.07±2.66	64.34±4.17	55.44±2.95	61.87±3.14
	Multi	64.14±3.17	66.39±3.32	66.72±2.82	60.21±3.72	64.54±2.36	65.08±2.39	63.71±2.88	63.13±2.58	55.04±3.15	61.89±2.93
Optimized	Single(w/o Atom and Mol)	60.01±2.94	58.08±5.85	61.36±2.38	52.62±4.80	60.00±2.89	59.98±3.56	58.78±2.79	57.16±4.63	53.85±1.87	57.61±3.05
	Single (w/o Mol)	60.23±2.84	61.26±4.44	61.59±5.40	53.32±4.06	60.19±2.81	61.70±3.60	59.76±3.09	58.34±4.50	53.58±1.66	58.20±3.10
	Single (w/o Atom)	60.71±2.91	59.71±4.76	60.88±4.17	51.16±3.98	59.81±3.27	59.80±3.24	59.33±4.20	56.62±4.32	54.12±2.50	57.73±3.40
	Single	61.07±2.66	59.62±4.40	61.96±4.85	53.09±4.18	60.99±3.50	61.06±3.60	59.78±3.31	58.23±4.25	54.90±1.77	58.61±3.17
	Multi(w/o Atom and Mol)	64.10±1.15	66.23±1.77	66.00±1.04	59.95±1.65	65.06±1.30	65.61±1.18	64.33±2.80	63.39±3.98	54.61±1.47	61.92±1.70
	Multi (w/o Mol)	63.27±1.47	68.11±2.44	67.15±1.83	60.93±1.99	64.26±1.61	65.52±1.33	62.73±1.57	63.83±3.86	55.78±1.34	62.04±1.71
	Multi (w/o Atom)	63.41±2.83	66.67±4.76	66.84±4.23	60.95±3.44	63.97±2.69	65.75±2.84	62.92±2.65	64.05±4.16	55.97±1.94	62.05±2.88
	Multi	63.51±1.17	67.44±1.33	67.23±1.81	61.35±1.73	63.66±2.38	65.56±2.40	62.89±1.17	64.85±2.88	56.13±1.17	62.18±1.64

		R-ACC									
Unoptimized	Single(w/o Atom and Mol)	93.42±2.34	92.18±4.10	93.59±2.70	93.57±3.65	93.99±2.51	94.20±2.10	93.99±2.71	88.91±5.80	94.29±1.41	93.56±2.56
	Single (w/o Mol)	93.34±2.61	91.89±5.11	93.18±3.67	93.79±2.70	94.41±2.19	94.49±2.94	94.10±2.34	89.29±6.68	94.24±1.24	93.63±2.67
	Single (w/o Atom)	93.37±2.22	91.86±3.20	93.26±3.03	93.05±4.88	94.16±2.43	94.26±3.11	93.54±4.18	88.91±6.28	94.26±1.34	93.44±2.92
	Single	93.57±2.55	91.75±4.03	93.47±2.71	93.78±3.16	94.37±3.18	94.31±2.35	93.81±2.39	89.10±5.30	94.47±1.34	93.66±2.57
	Multi(w/o Atom and Mol)	93.86±1.95	93.04±2.97	94.78±2.37	94.31±1.99	95.17±1.59	95.76±1.93	94.34±2.07	89.61±3.12	94.12±1.76	94.15±2.03
	Multi (w/o Mol)	93.71±1.50	93.16±3.02	94.60±1.83	94.49±1.76	95.14±1.45	95.84±1.44	94.38±1.58	89.49±2.75	94.28±1.62	94.18±1.72
	Multi (w/o Atom)	93.69±1.63	92.98±2.90	94.57±2.19	94.39±2.18	95.31±1.66	95.74±1.49	94.48±1.61	89.82±3.31	94.28±1.62	94.20±1.85
	Multi	93.76±1.30	93.14±2.58	94.59±1.69	94.41±1.85	95.24±1.30	95.82±1.36	94.61±1.50	89.46±2.92	94.37±1.37	94.24±1.58
Optimized	Single(w/o Atom and Mol)	93.41±2.57	91.81±4.82	93.01±2.29	93.78±3.73	94.22±2.11	94.42±2.79	94.15±3.35	89.12±5.76	94.27±1.41	93.60±2.72
	Single (w/o Mol)	93.41±2.47	92.37±3.70	93.53±2.98	93.39±4.25	94.08±1.69	94.20±2.91	93.89±3.82	89.64±5.48	94.48±1.38	93.64±2.74
	Single (w/o Atom)	93.49±2.22	91.51±3.20	92.99±3.03	93.57±4.88	94.63±2.43	94.35±3.11	94.19±4.18	89.24±6.28	94.24±1.35	93.62±2.93
	Single	93.64±3.07	92.06±3.88	93.22±2.62	93.85±4.95	94.62±2.43	94.76±3.00	94.33±4.02	88.96±5.34	94.43±1.19	93.80±2.93
	Multi(w/o Atom and Mol)	93.77±1.34	93.28±2.06	94.53±1.36	94.56±1.31	95.17±1.03	95.81±1.14	94.43±1.26	89.83±3.02	94.43±1.20	94.26±1.37
	Multi (w/o Mol)	93.97±1.14	92.96±1.97	94.87±1.35	94.61±1.48	95.40±1.20	95.84±1.28	94.65±1.26	89.47±2.75	94.24±1.17	94.29±1.36
	Multi (w/o Atom)	93.94±1.41	92.79±2.21	94.83±1.68	94.53±1.37	95.36±0.96	95.87±1.34	94.55±1.26	89.34±3.38	94.23±1.30	94.24±1.47
	Multi	93.99±0.98	93.15±1.84	94.89±1.34	94.69±1.34	95.36±1.13	95.90±1.23	94.59±0.97	89.95±2.92	94.24±1.02	94.33±1.24
		A-ACC									
Unoptimized	Single(w/o Atom and Mol)	92.78±2.35	92.36±4.10	92.74±2.72	93.11±3.65	93.55±2.51	93.52±2.10	93.74±2.72	91.25±5.80	93.67±1.41	93.21±2.57
	Single (w/o Mol)	92.88±2.63	92.53±5.13	93.01±3.67	93.34±2.71	93.85±2.19	93.66±2.95	93.90±2.35	91.16±6.68	93.79±1.25	93.37±2.68
	Single (w/o Atom)	92.95±2.22	92.58±3.20	93.19±3.04	92.87±4.88	94.01±2.43	93.75±3.11	93.56±4.18	90.81±6.28	93.94±1.35	93.36±2.93
	Single	93.10±2.56	92.36±4.03	93.37±2.72	93.39±3.16	94.13±3.18	93.77±2.35	93.69±2.39	90.92±5.31	94.20±1.35	93.52±2.58
	Multi(w/o Atom and Mol)	93.48±1.95	93.95±2.97	94.61±2.38	94.00±1.99	94.70±1.60	94.97±1.93	94.05±2.08	92.30±3.13	93.59±1.77	93.95±2.03
	Multi (w/o Mol)	93.31±1.50	94.03±3.03	94.49±1.83	94.34±1.77	94.66±1.45	95.14±1.45	94.24±1.59	91.92±2.75	93.85±1.63	94.03±1.72
	Multi (w/o Atom)	93.31±1.64	93.74±2.90	94.41±2.19	94.45±2.19	94.95±1.67	95.10±1.49	94.38±1.62	92.03±3.31	93.94±1.62	94.10±1.86
	Multi	93.42±1.32	93.75±2.59	94.45±1.69	94.39±1.85	94.94±1.30	95.13±1.37	94.52±1.51	91.88±2.92	94.05±1.37	94.15±1.58
Optimized	Single(w/o Atom and Mol)	92.68±2.57	92.77±4.83	93.37±2.29	93.00±3.73	93.47±2.12	93.47±2.79	93.73±3.36	90.93±5.76	93.70±1.42	93.23±2.73
	Single (w/o Mol)	92.85±2.47	92.92±3.71	93.30±2.98	93.05±4.25	93.75±1.69	93.59±2.91	93.81±3.82	91.10±5.48	93.99±1.38	93.40±2.74
	Single (w/o Atom)	93.11±2.23	92.35±3.20	93.00±3.04	93.26±4.89	94.22±2.43	93.55±3.12	93.98±4.19	91.37±6.29	93.84±1.36	93.45±2.93
	Single	93.17±3.08	92.72±3.88	93.14±2.63	93.44±4.96	94.16±2.44	94.12±3.01	94.09±4.03	91.39±5.35	94.14±1.19	93.65±2.93
	Multi(w/o Atom and Mol)	93.32±1.34	93.90±2.07	94.42±1.37	94.30±1.31	94.69±1.04	95.14±1.14	94.16±1.27	92.15±3.03	93.90±1.21	94.04±1.38
	Multi (w/o Mol)	93.56±1.15	94.12±1.97	94.72±1.35	94.40±1.49	94.89±1.21	95.07±1.29	94.37±1.26	92.54±2.76	93.79±1.18	94.15±1.37
	Multi (w/o Atom)	93.60±1.42	93.80±2.22	94.66±1.69	94.40±1.37	94.95±0.97	95.11±1.34	94.41±1.27	92.26±3.39	93.89±1.30	94.16±1.48
	Multi	93.65±0.98	93.80±1.85	94.65±1.35	94.44±1.35	95.01±1.13	95.06±1.24	94.48±0.98	92.20±2.92	93.97±1.03	94.20±1.25

Top-1

	Single(w/o Atom and Mol)	65.20±4.78	67.92±9.05	62.38±6.67	57.12±7.80	59.19±5.80	63.70±6.60	64.75±5.60	64.15±7.63	58.44±3.29	61.90±5.56
	Single (w/o Mol)	67.42±4.17	63.80±7.89	59.58±6.54	57.69±8.28	63.58±6.50	63.39±6.21	64.57±4.29	63.44±7.97	57.26±3.86	61.97±5.46
	Single (w/o Atom)	67.46±5.44	64.21±6.99	60.65±6.61	58.16±7.72	60.18±6.39	65.17±6.85	64.39±5.52	64.25±8.05	55.96±3.35	61.60±5.67
Unoptimized	Single	68.05±4.41	65.02±7.39	60.80±7.68	58.31±7.82	61.90±6.22	64.16±6.04	66.20±3.99	64.16±8.30	57.46±3.36	62.45±5.32
	Multi(w/o Atom and Mol)	66.32±4.62	68.12±7.38	68.39±7.15	65.35±7.13	64.50±5.81	67.27±5.78	66.91±4.54	65.39±7.14	59.28±3.67	64.63±5.27
	Multi (w/o Mol)	68.30±4.96	70.50±9.14	69.61±6.94	63.96±6.79	66.36±6.06	69.01±5.72	67.38±4.98	67.17±6.40	58.89±3.73	65.49±5.43
	Multi (w/o Atom)	67.41±4.95	67.24±7.26	69.03±5.74	63.69±7.11	64.32±6.57	68.43±5.65	67.29±4.04	66.47±7.06	57.92±4.37	64.55±5.39
	Multi	68.46±4.84	67.95±8.40	70.56±7.21	64.48±7.65	67.66±5.96	69.17±5.16	68.49±4.09	66.65±6.04	57.95±3.85	65.54±5.26
	Single(w/o Atom and Mol)	67.28±5.00	63.84±8.99	62.06±7.74	59.17±6.37	61.29±5.79	61.94±6.69	64.30±5.24	64.84±8.32	57.75±4.19	61.97±5.79
	Single (w/o Mol)	65.91±4.49	66.60±8.88	63.70±6.65	57.35±7.13	62.10±6.59	64.15±6.94	65.45±5.03	64.02±7.27	57.11±3.44	62.18±5.53
	Single (w/o Atom)	67.72±5.44	64.44±6.99	61.79±6.60	59.20±7.72	60.08±6.39	62.57±6.85	64.61±5.51	63.76±8.05	58.23±4.69	62.08±6.00
Optimized	Single	67.61±4.88	66.63±8.64	63.75±6.40	59.15±7.88	59.83±5.81	64.84±6.52	65.27±5.79	64.89±7.95	58.05±4.18	62.63±5.80
	Multi(w/o Atom and Mol)	67.91±4.29	69.56±8.53	68.73±7.24	62.84±6.78	65.80±5.74	67.98±4.95	67.54±4.87	67.96±6.87	57.48±3.39	64.79±5.13
	Multi (w/o Mol)	67.79±4.19	70.32±8.94	69.25±6.07	65.31±7.40	64.85±5.61	69.44±4.58	67.88±4.60	67.94±6.28	59.74±3.73	65.68±5.05
	Multi (w/o Atom)	67.83±5.20	67.67±6.43	69.70±6.38	64.36±5.85	64.95±6.06	69.23±5.81	67.06±3.25	66.90±8.73	59.62±3.34	65.31±5.04
	Multi	69.32±3.58	67.53±8.03	70.64±6.50	64.95±7.36	65.79±4.83	68.26±4.19	68.27±4.97	67.00±7.38	59.49±3.39	65.75±4.84
Top-2											
	Single(w/o Atom and Mol)	83.89±4.01	78.57±7.64	79.14±6.25	76.05±7.33	82.33±4.76	82.49±4.57	82.03±4.66	80.62±6.10	76.60±2.99	80.15±4.68
	Single (w/o Mol)	82.97±3.49	82.93±6.34	82.01±5.82	77.63±5.85	81.92±4.99	83.26±4.99	81.88±4.10	81.32±5.22	76.41±2.75	80.55±4.30
	Single (w/o Atom)	84.78±4.49	79.36±6.24	79.68±5.96	77.04±6.43	81.04±4.69	83.16±5.10	82.46±4.79	81.63±6.29	75.84±2.66	80.28±4.60
Unoptimized	Single	85.59±3.13	81.97±6.80	81.33±6.15	77.60±6.06	81.17±5.76	83.55±4.70	80.84±4.02	81.23±6.38	77.39±2.42	80.87±4.34
	Multi(w/o Atom and Mol)	85.93±3.42	87.60±5.66	87.12±5.10	83.64±5.52	84.08±4.33	86.19±3.26	84.60±3.81	86.59±5.35	76.04±3.82	83.12±4.15
	Multi (w/o Mol)	86.02±3.53	89.93±5.19	87.10±4.73	85.34±4.88	85.34±3.57	87.47±4.79	85.38±3.66	86.83±5.26	78.62±3.16	84.41±3.96
	Multi (w/o Atom)	85.59±3.26	88.50±5.82	87.02±5.13	84.58±4.95	84.40±3.63	87.16±3.53	85.39±4.08	87.55±4.77	76.64±3.49	83.63±3.96
	Multi	86.02±3.28	88.01±6.30	88.29±5.22	84.25±5.17	85.15±4.05	86.87±3.08	86.09±3.86	88.04±5.86	76.81±3.33	83.95±4.01
	Single(w/o Atom and Mol)	85.26±4.36	79.73±7.57	80.56±5.92	77.98±6.11	81.94±5.38	82.30±4.13	81.39±4.23	79.56±6.16	77.85±3.35	80.74±4.68
	Single (w/o Mol)	84.19±3.47	83.26±6.48	80.03±5.04	77.83±5.76	80.54±5.36	82.43±4.61	81.45±4.80	82.04±5.90	77.96±3.08	80.74±4.46
	Single (w/o Atom)	85.65±4.50	81.06±6.24	79.88±5.95	77.97±6.42	80.67±4.68	82.25±5.10	81.45±4.79	79.91±6.29	76.79±3.70	80.42±4.85
Optimized	Single	85.05±4.11	80.90±7.28	80.80±5.41	76.28±5.87	82.50±4.67	83.52±4.12	82.59±5.60	82.02±5.41	77.04±2.99	80.96±4.55
	Multi(w/o Atom and Mol)	85.78±3.46	87.86±5.89	86.64±6.06	85.21±4.76	84.89±4.67	86.05±3.64	84.37±3.50	86.32±4.92	78.08±3.02	83.73±3.99
	Multi (w/o Mol)	86.19±2.82	91.10±5.82	89.01±4.92	84.66±4.66	86.05±3.79	87.20±3.59	85.76±3.14	88.59±4.98	77.44±2.59	84.49±3.55
	Multi (w/o Atom)	85.47±3.87	87.94±5.46	85.99±4.80	85.12±4.76	84.75±4.06	86.71±3.79	85.56±3.24	85.97±5.53	78.28±2.97	83.88±3.88
	Multi	86.95±2.89	86.75±5.13	87.39±5.10	85.85±5.46	85.54±4.02	87.86±3.01	85.85±2.99	87.24±5.37	78.28±3.34	84.52±3.75
Jaccard											

Unoptimized	Single(w/o Atom and Mol)	44.98±5.63	40.96±6.51	43.28±5.82	38.91±5.99	45.37±5.26	45.80±4.70	44.86±6.05	41.50±7.77	39.68±2.81	42.83±5.05
	Single (w/o Mol)	45.56±5.22	41.86±8.16	44.35±6.97	40.99±5.17	46.12±5.01	46.93±6.28	45.10±5.55	42.58±8.95	39.94±2.34	43.55±5.13
	Single (w/o Atom)	45.77±4.49	43.48±6.32	43.30±6.27	38.73±7.17	45.72±5.16	46.22±6.47	45.10±7.29	42.18±6.81	39.81±2.59	43.24±5.24
	Single	45.71±5.15	43.61±6.89	43.90±6.05	38.45±5.00	45.30±6.47	46.91±5.09	46.02±5.78	41.47±8.05	39.78±2.90	43.37±5.14
	Multi(w/o Atom and Mol)	48.05±4.25	43.39±7.05	45.30±5.66	47.11±4.30	46.96±4.25	45.67±5.19	48.09±4.23	47.27±6.25	41.07±3.27	45.39±4.47
	Multi (w/o Mol)	47.56±4.03	44.68±6.90	46.33±5.05	47.21±5.03	46.99±4.25	46.70±4.56	47.19±3.72	47.70±5.84	41.24±3.00	45.52±4.20
	Multi (w/o Atom)	47.93±4.07	43.56±6.92	46.70±5.67	45.86±5.00	47.34±4.70	46.77±4.61	48.72±4.04	47.90±6.63	40.99±3.28	45.66±4.47
	Multi	47.88±4.21	43.67±7.11	45.83±5.21	47.23±4.85	48.09±4.32	46.65±4.46	47.87±4.13	48.62±6.04	40.82±2.75	45.65±4.25
Optimized	Single(w/o Atom and Mol)	45.55±5.42	40.96±8.14	45.35±6.12	40.99±6.27	44.51±5.10	45.96±6.36	44.45±6.82	40.86±7.22	40.83±3.26	43.31±5.49
	Single (w/o Mol)	45.05±5.24	43.93±6.04	45.27±6.93	40.48±7.31	45.73±4.75	46.57±6.27	44.80±7.73	42.88±6.78	40.92±2.95	43.74±5.46
	Single (w/o Atom)	45.07±4.49	43.00±6.32	45.05±6.27	39.01±7.17	44.86±5.16	46.92±6.47	44.10±7.29	41.17±6.81	40.63±3.20	43.25±5.39
	Single	45.32±6.16	43.28±6.92	45.71±6.38	39.44±7.97	44.44±5.01	46.64±6.12	44.26±7.70	41.16±6.51	40.90±2.64	43.39±5.56
	Multi(w/o Atom and Mol)	48.29±3.75	44.90±5.35	47.91±6.16	46.00±4.60	47.37±3.96	47.23±4.48	47.88±4.04	47.98±4.98	40.06±3.36	45.58±4.18
	Multi (w/o Mol)	48.63±3.19	45.79±5.17	48.87±4.89	47.03±4.17	49.44±3.43	48.16±4.39	48.79±3.69	49.54±4.70	40.66±3.23	46.51±3.80
	Multi (w/o Atom)	48.15±4.11	44.78±6.01	47.98±5.32	45.64±4.43	47.94±3.63	47.72±4.32	48.55±3.46	47.65±6.02	40.12±3.46	45.73±4.14
	Multi	48.66±3.29	44.10±3.75	47.36±5.08	46.36±4.51	48.98±3.97	48.36±4.38	48.77±3.18	47.90±4.42	40.72±2.91	46.17±3.68

*Single and Multi denote single-task and multi-task Graph Neural Network (GNN) architectures, respectively.

Atom and Mol refer to the integration of atomic-level reactivity descriptors and global molecular-level properties; w/o indicates the exclusion of these specific multi-scale features in ablation studies.

Optimized and Unoptimized indicate the data splitting strategies employed during cross-validation. To address the complexity of substrate molecules undergoing multiple concurrent transformation types, the Optimized approach utilizes a frequency-aware hierarchical stratification. Substrates are classified for splitting based on their rarest reaction type, adhering to the following priority hierarchy: Reduction > Rearrangement > C-O Oxidation > Epoxidation > Heter-Oxidation > Dealkylation > Hydroxylation. This systemic prioritization prevents data leakage and ensures consistent, balanced partitioning of minority metabolic reactions across folds.

W*avg denotes the weighted average performance across all nine CYP450 isoforms.

Metrics is comprehensively evaluated from two distinct perspectives:

(1) A (All/Absolute perspective) pools all potential reactive sites across the entire dataset, effectively measuring the model's global capability to distinguish metabolic from non-metabolic sites.

(2) R (Ranking perspective) evaluates performance on a strictly per-molecule basis, assessing the model's practical utility in correctly ranking the true metabolic sites higher than non-metabolic sites within each individual substrate. W*avg denotes the weighted average performance across all nine CYP450 isoforms.

REFERENCES

1. Yang,K., Swanson,K., Jin,W., Coley,C., Eiden,P., Gao,H., Guzman-Perez,A., Hopper,T., Kelley,B., Mathea,M., *et al.* (2019) Analyzing Learned Molecular Representations for Property Prediction. *J. Chem. Inf. Model.*, **59**, 3370–3388.

2. Heid,E., Greenman,K.P., Chung,Y., Li,S.-C., Graff,D.E., Vermeire,F.H., Wu,H., Green,W.H. and McGill,C.J. (2024) Chemprop: A Machine Learning Package for Chemical Property Prediction. *J. Chem. Inf. Model.*, **64**, 9–17.

3. Battaglia,P.W., Hamrick,J.B., Bapst,V., Sanchez-Gonzalez,A., Zambaldi,V., Malinowski,M., Tacchetti,A., Raposo,D., Santoro,A., Faulkner,R., *et al.* (2018) Relational inductive biases, deep learning, and graph networks. 10.48550/arXiv.1806.01261.

4. Gilmer,J., Schoenholz,S.S., Riley,P.F., Vinyals,O. and Dahl,G.E. (2017) Neural Message Passing for Quantum Chemistry. 10.48550/arXiv.1704.01212.

5. Lakshminarayanan,B., Pritzel,A. and Blundell,C. (2017) Simple and Scalable Predictive Uncertainty Estimation using Deep Ensembles. 10.48550/arXiv.1612.01474.

6. Hirschfeld,L., Swanson,K., Yang,K., Barzilay,R. and Coley,C.W. (2020) Uncertainty Quantification Using Neural Networks for Molecular Property Prediction. *J. Chem. Inf. Model.*, **60**, 3770–3780.

7. Scalia,G., Grambow,C.A., Pernici,B., Li,Y.-P. and Green,W.H. (2020) Evaluating Scalable Uncertainty Estimation Methods for Deep Learning-Based Molecular Property Prediction. *ACS*.
